# Host Cell Restriction Factors of Bunyaviruses and Viral Countermeasures

**DOI:** 10.3390/v13050784

**Published:** 2021-04-28

**Authors:** Solène Lerolle, Natalia Freitas, François-Loïc Cosset, Vincent Legros

**Affiliations:** 1 CIRI—Centre International de Recherche en Infectiologie, Université de Lyon, Université Claude Bernard Lyon 1, Inserm, U1111, CNRS, UMR5308, ENS Lyon, 46 allée d’Italie, 69007 Lyon, France; solene.lerolle@ens-lyon.fr (S.L.); natalia.bezerra-de-freitas@ens-lyon.fr (N.F.); vincent.legros@ens-lyon.fr (V.L.); 2 Université de Lyon, VetAgro Sup, 69280 Marcy-l’Étoile, France

**Keywords:** bunyaviruses, restriction factors, innate immunity, interferon, viral countermeasures

## Abstract

The *Bunyavirales* order comprises more than 500 viruses (generally defined as bunyaviruses) classified into 12 families. Some of these are highly pathogenic viruses infecting different hosts, including humans, mammals, reptiles, arthropods, birds, and/or plants. Host cell sensing of infection activates the innate immune system that aims at inhibiting viral replication and propagation. Upon recognition of pathogen-associated molecular patterns (PAMPs) by cellular pattern recognition receptors (PRRs), numerous signaling cascades are activated, leading to the production of interferons (IFNs). IFNs act in an autocrine and paracrine manner to establish an antiviral state by inducing the expression of hundreds of IFN-stimulated genes (ISGs). Some of these ISGs are known to restrict bunyavirus infection. Along with other constitutively expressed host cellular factors with antiviral activity, these proteins (hereafter referred to as “restriction factors”) target different steps of the viral cycle, including viral entry, genome transcription and replication, and virion egress. In reaction to this, bunyaviruses have developed strategies to circumvent this antiviral response, by avoiding cellular recognition of PAMPs, inhibiting IFN production or interfering with the IFN-mediated response. Herein, we review the current knowledge on host cellular factors that were shown to restrict infections by bunyaviruses. Moreover, we focus on the strategies developed by bunyaviruses in order to escape the antiviral state developed by the infected cells.

## 1. Introduction

Viral infections are often detrimental to the host, disrupting their homeostasis and jeopardizing their survival. In reaction to this, hosts have developed several strategies to recognize viral pathogens and restrict their replication. Restriction factors represent crucial factors of the host intrinsic and innate immunity involved in antagonizing viral invasion and replication. This set of proteins, whether constitutively expressed or induced by the interferons (IFNs), are key factors of the innate immune response and utilize diverse mechanisms to inhibit specific steps of the viral life cycle: entry, replication, transcription, translation and/or egress. However, viruses enter into an arms race to escape host immunity by directing some of their proteins to counteract these restriction factors in a more or less specific manner.

The *Bunyavirales* order comprises a large number of emerging, medically and/or economically important viruses known as bunyaviruses. The molecular biology of bunyaviruses is an emerging field of active research. In recent years, numerous studies have focused on proteins restricting the bunyavirus life cycle and on their viral countermeasures. In this review, we aim at discussing the restriction factors which have been shown to inhibit bunyaviruses replication at distinct stages of their life cycles, including viral entry, replication and egress. Additionally, the strategies developed by these viruses to circumvent and limit the impact of these restriction factors are underlined.

## 2. Medical Importance of Viruses in the *Bunyavirales* Order

The *Bunyavirales* order was established in 2017 and contains more than 500 viruses classified into 12 families. Previously, viruses from the *Bunyavirales* order were classified into two families: the *Bunyaviridae* and the *Arenaviridae* families. However, structural and functional similarities between members of the two families led to the establishment of the new *Bunyavirales* order, containing viruses with segmented, linear, single-stranded, negative-sense or ambisense RNA genomes [1]. Notably, five families within the *Bunyavirales* order (*Arenaviridae*, *Hantaviridae*, *Nairoviridae*, *Peribunyaviridae* and *Phenuiviridae*, on which we focus in this review, as listed in Table 1) contain viruses of medical or economic importance, as they are able to cause diseases in mammalian hosts (including humans and livestock) as well as in plants.

Members of the *Arenaviridae* family (arenaviruses), are associated with rodent-transmitted disease in humans. They are commonly divided into Old-World and New-World arenaviruses. Notably, Lassa virus (LASV), Junín virus (JUNV), Machupo virus (MACV), Guanarito virus (GTOV) or Sabiá virus (SBAV) are responsible for viral hemorrhagic fevers in humans. With a prevalence of 300,000 cases per year, LASV is the most prevalent and threatening [2]. The prototypic Old-World arenavirus lymphocytic choriomeningitis virus (LCMV) is responsible for a mild febrile illness that can develop to a neurological disease. Like arenaviruses, members of the *Hantaviridae* family (hantaviruses) are also transmitted by rodents to humans. They are responsible for two types of diseases in humans: the hemorrhagic fever with renal syndrome (HFRS), and the hantavirus cardio-pulmonary syndrome (HCPS). Notably, Hantaan virus (HTNV) and Seoul virus (SEOV) are of significant medical importance, with mortality rates reaching up to 15% [3]. The *Nairoviridae* family contains tick-transmitted viruses (nairoviruses) of both medical and economic importance. Some are human pathogens, with the most important to date being the Crimean-Congo hemorrhagic fever virus (CCHFV), which is responsible for a viral hemorrhagic fever with a mortality rate reaching 30%. Others are pathogenic to livestock, and are therefore at the origin of important economic losses, such as Nairobi sheep disease virus (NSDV) [4]. In the *Peribunyaviridae* family are arboviruses (peribunyaviruses) which are also responsible for diseases in humans and in livestock. In humans, Oropouche virus (OROV) or La Crosse virus (LACV) are responsible for fever-like symptoms that can lead to an encephalitis. In livestock, Schmallenberg virus (SBV) is notably responsible for abortions and newborn malformations, leading to important economic losses in endemic regions. Bunyamwera virus (BUNV) is considered the prototype virus of this family [5]. Finally, members of the *Phenuiviridae* family are arboviruses (phenuiviruses) such as the Rift valley fever virus (RVFV) or the severe fever with thrombocytopenia syndrome virus (SFTSV). RVFV is a mosquito-borne virus responsible for abortions and important economic losses in livestock, as well as for febrile illness in humans. Hemorrhagic fevers were additionally described in 1% of the RVFV infection cases in humans [6].

This non-exhaustive list of diseases caused by bunyaviruses reveals the economic and medical importance of these viruses. The absence of etiologic treatments or prophylactic strategies against some, such as CCHFV and LASV, makes the research on these viruses a priority. Thus, it is of significant importance to increase the research on bunyaviruses to understand their pathogenicity and to pave the way to new treatments and vaccines. To this end, three diseases caused by bunyaviruses, namely Crimean-Congo hemorrhagic fever, Lassa fever and Rift valley fever, are listed among the nine medically and economically important diseases registered in the R&D Blueprint program of the World Health Organization (WHO), which aims at prioritizing the research in public health emergency contexts [7].

## 3. Morphology of Bunyavirus Particles and Genome Organization

**Figure 1:**
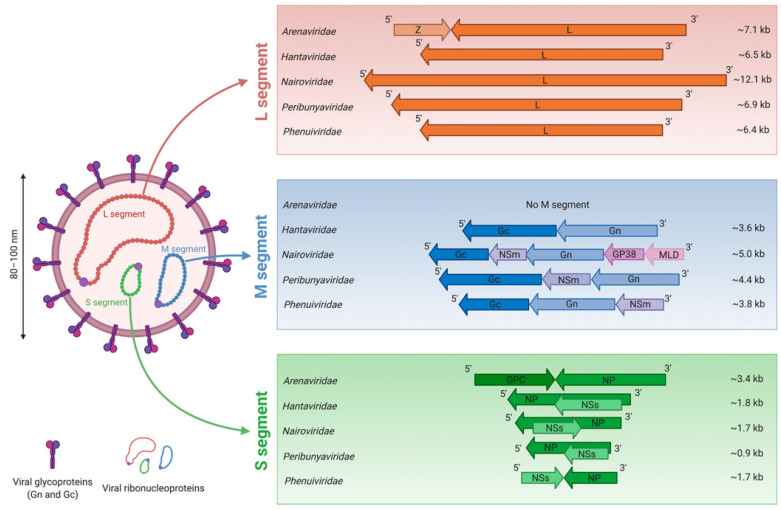
Schematic representation of viral genomes from the *Bunyavirales* order. A typical bunyaviral particle is represented on the left. The particularities of the genome from each family are represented on the right. The nucleotide lengths are displayed and based on full-length sequences available in GenBank. The viral M segment encodes a glycoprotein precursor (GPC) (not represented in the figure), which is further matured into different proteins (Gn, Gc, and sometimes NSm, MLD, GP38) that are depicted here on the viral genome. GPC: glycoproteins precursor; NP: nucleoprotein; NSm: non-structural protein M; NSs: non-structural protein S; MLD: mucin-like domain; Z: zinc-binding matrix protein.

Typical bunyavirus particles have a lipid envelope containing two viral glycoproteins, termed Gn and Gc. The viral negative-sense RNA genome is tri-segmented, and the three segments are termed according to their size: small (S), medium (M) and large (L) segment [8,9,10] (Figure 1). Unusually, arenaviruses have a bi-segmented RNA genome with only the S and L segments [11].

The S segment encodes the nucleoprotein (NP) involved in the formation of viral ribonucleoparticles (RNPs) [12]. Moreover, the S segment of some peribunyaviruses and hantaviruses also has an overlapping functional open reading frame (ORF) which encodes a non-structural protein termed NSs as the result of alternate AUG-initiation codon selection [8,13]. Some phenuiviruses and nairoviruses’ S segments have the particularity to encode a protein called NSs in the opposite orientation relative to the NP gene, in an ambisense strategy [14,15]. The ORF of CCHFV NSs overlaps the NP ORF which is in contrast to that of phenuiviruses, where the viral proteins NP and NSs coding regions are separated by an intergenic region.

The M segment encodes the glycoproteins precursor (GPC), which is ultimately matured as Gn and Gc glycoproteins. Additionally, for some viruses, the maturation of the GPC leads to the maturation of non-structural proteins, such as NSm for RVFV, NSm for some peribunyaviruses, and NSm and GP38 associated to a mucin-like domain (MLD) for CCHFV [5,16].

Finally, the L segment encodes a unique protein, the L protein, which possesses the RNA-dependent RNA polymerase (RdRP) activity [8,9,10].

As previously mentioned, arenaviruses have only two segments, termed S and L. Both segments encode two genes in an ambisense orientation separated by an intergenic region [17]. The S segment encodes the NP and the GPC, which is further matured into the glycoproteins Gn and Gc. In addition to the L protein, the L segment encodes a zinc-binding matrix protein termed Z protein [11].

## 4. Bunyavirus Cell Cycle

Despite the high number of virus species in the *Bunyavirales* order, their cycles share similarities. The typical bunyaviruses cell cycle is briefly described here [3,10]. The viral cycle is initiated upon the interaction between one or two glycoproteins located at the surface of the virions with a cellular receptor. As far as we know, entry of all bunyaviruses into cells is mediated by endocytosis. Following fusion between virion and endosomal membranes, the viral genome is liberated into the cytoplasm. The protein L, which is part of the RNPs and contains a RdRP domain, uses the segments as templates for the production of messenger RNAs (mRNAs), which are translated into proteins thanks to the cellular translation machinery. Bunyaviruses have developed a cap-snatching mechanism, by which they steal the cap of cellular mRNAs in order to initiate the translation of viral mRNAs [18]. Replication of the viral genome also is ensured by the RdRP domain of the L protein. Viral genomic RNA is used as a template to generate a complementary RNA intermediate known as antigenomic RNA, which is then encapsidated by the NP. These antigenomic RNPs are later used as a template for the synthesis of new genomic RNPs. Once the new RNPs and viral proteins are synthetized, assembly and budding occurs at membranes of the Golgi apparatus. New virions are thought to leave the infected cell by exocytosis.

## 5. Induction of Type I Interferons and Innate Immunity Following Bunyavirus Infection

Upon infection, pathogens and their molecular associated patterns (PAMPs) are recognized by a large variety of cellular proteins known as pattern recognition receptors (PRRs) [19,20]. PRRs can be found in the cytoplasm, such as in the family of the retinoic acid-inducible gene-I (RIG-I)-like receptors (RLRs) and the family of nucleotide-binding oligomerization domain (NOD)-like receptors (NLRs). More precisely, two RNA helicases among the RLR family, RIG-I and the melanoma differentiation-associated protein 5 (MDA5), are the main intracellular receptors of viral RNA. Indeed, RIG-I is activated upon binding to short double-stranded (ds) RNA molecules and to the triphosphate group at the 5′ end of viral single-stranded (ss) RNA, while MDA5 activation is dependent on longer dsRNA structures [20,21]. Upon activation of these PRRs, signaling cascades are triggered, involving the activation of TANK-binding kinase 1 (TBK1) and IκB kinase-ε (IKKε), which in turn phosphorylate the IFN regulatory factors 3 and 7 (IRF3 and 7). IRF3 and IRF7 then translocate into the nucleus to induce the transcription of genes encoding type I and type III IFN [20], key factors of the innate immunity.

The activation of RIG-I is at the origin of the production of type I IFN upon infection of some bunyaviruses such as JUNV [22] and SFTSV [23]. Moreover, isolated genomic RNAs from particles of LASV or RVFV strongly activate the production of type I IFN in a RIG-I-dependent manner [24]. Conversely, genomic RNAs isolated from CCHFV and HTNV particles do not induce a RIG-I-dependent IFN production. Notably, the genomic RNAs of both viruses are monophosphorylated at their 5′ end, instead of having a tri-phosphorylated 5′ end (5′ppp). Thus, suggestions were made that the 5′ppp ssRNA of bunyaviruses genomes are the PAMPs recognized by RIG-I. Additional data identified NP associated with a 5′ppp dsRNA panhandle, as seen in the genome of bunyaviruses, as a potent RIG-I activator [25]. Moreover, it was shown that despite having a 5′p end, HTNV replication is still impaired by RIG-I, and that HTNV NP stimulates RIG-I signaling [26]. As seen in HTNV, another study showed that CCHFV still activates RIG-I despite its modified 5′ end [27]. However, the implication of CCHFV NP as component of a putative PAMP recognized by RIG-I was not assessed and further studies are warranted to decipher the nature of CCHFV’s RIG-I-activating PAMP.

MDA5 also is implicated in the detection of bunyaviruses, like LCMV, as shown by in vitro studies and in vivo studies in MDA5-knock-out (KO) mice. LCMV RNA was sufficient to induce in vitro production of type I IFN and interacts with MDA5, defining it as putative PAMP [28]. However, live LCMV did not induce production of type I IFN, suggesting that other LCMV components than LCMV RNA interfere with the production of type I IFN, as later discussed in this review.

Other PRRs are also found at membranes, such as the plasma or endosomal membranes, like the family of Toll-like receptors (TLRs). TLR3 implication in the induction of IFN-mediated responses upon HTNV infection was shown in vitro but remains to be confirmed in vivo [29]. Another TLR, TLR7, is implicated in LCMV-induced production of type I IFN in plasmacytoid dendritic cells [30]. Finally, JUNV glycoproteins were found to activate TLR2-mediated innate immune responses in vitro [31].

After their production, type I and type III IFN are secreted and act in an autocrine and paracrine manner upon recognition by their receptors (IFN-α/β receptor (IFNAR1/2) and IFN-λ receptor 1/interleukin-10 receptor 2 (IFNLR1/IL10R2) respectively), through the activation of the janus kinases/signal transducer and activator of transcription proteins (JAK/STAT) pathway. Binding of these IFNs to their cognate receptors triggers phosphorylation of janus kinase 1 (JAK1) and tyrosine kinase 2 (TYK2), which leads to the phosphorylation and association in a heterodimer of signal transduced and activator of transcription proteins 1 and 2 (STAT1 and 2). After the recruitment of IRF9 and translocation into the nucleus, this heterodimer activates the transcription of IFN-stimulated genes (ISGs), with antiviral, immunomodulatory and antiproliferative properties (reviewed in [32]). The ISGs induced by type III IFN overlap those induced by type I IFN, with type I IFN acting in a more potent and rapid manner [33].

## 6. Inhibition of Bunyavirus by IFN-Stimulated Genes and Other Antiviral Factors

**Figure 2:**
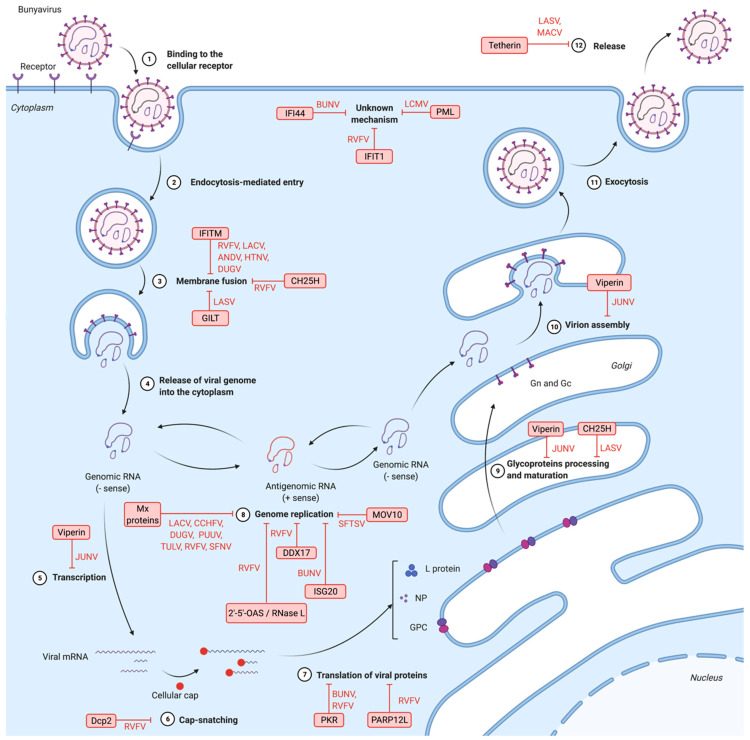
Steps of the bunyavirus cycle inhibited by host restriction factors. Bunyaviruses bind to the receptor (1), then enter the cell and release their genome into the cytoplasm (2, 3 and 4). Genomic RNA is then transcribed into mRNA (5 and 6), later translated into viral proteins (7 and 9). Moreover, genomic RNA is replicated (8). New virions assemble and exit the cell (10, 11 and 12). Each step of the cycle can be inhibited by host restriction factors, which are written in red boxes. ANDV: Andes virus; BUNV: Bunyamwera virus; CCHFV: Crimean-Congo hemorrhagic fever virus; DUGV: Dugbe virus; HTNV: Hantaan virus; JUNV: Junín virus; LACV: La Crosse virus; LASV: Lassa virus; LCMV: lymphocytic choriomeningitis virus; MACV: Machupo virus; PUUV: Puumala virus; RVFV: Rift Valley fever virus; SFNV: Sandfly fever Naples virus; SFTSV: severe fever with thrombocytopenia syndrome virus; TULV: Tula virus. 2′-5′-OAS: 2′-5′-oligoadenylate synthetase; CH25H: cholesterol-25-hydrolase; Dcp2: Decapping 2; IFI44: interferon (IFN)-induced protein 44; IFIT1: IFN-induced protein with tetratricopeptide repeats 1; IFITM: IFN-induced transmembrane protein; ISG20: IFN-stimulated gene 20; GILT: γ-IFN-inducible lysosomal thiol reductase; MOV10: Moloney leukemia virus 10 protein; Mx: myxovirus resistance protein; PARP12L: long isoform of poly(ADP-ribose) polymerase 12; PKR: protein kinase R; PML: promyelocytic leukemia protein.

Upon bunyavirus infection and sensing by the PRRs, type I IFNs are produced. The importance of the induction of type I IFN synthesis has been shown for some bunyaviruses, like CCHFV, LACV, BUNV, HTNV, RVFV or Punta Toro virus (PTV). Indeed, for these viruses, infection of IFN-deficient animal models leads to the development of a severe disease, while the infection of immunocompetent animal models is less severe or even asymptomatic [34,35,36,37,38,39,40,41]. This is a solid evidence for the importance of the IFN-induced antiviral state against bunyaviruses, suggesting that some ISGs are restriction factors for bunyaviruses. Recently, a mouse-adapted variant of CCHFV that recapitulates the disease in immunocompetent mice was reported [42], but how the mutations found in this variant enable the virus to escape the immune system of the immunocompetent mice remains to be characterized. Moreover, bunyavirus infection induces the upregulation of the expression of several known ISGs, as shown by different transcriptome analyses performed on RVFV-infected human kidney or airway epithelial cells [43,44], on SBV-infected primary bovine fibroblasts [45], or on LASV-exposed human or non-human primate peripheral blood mononuclear cells [46,47]. In addition to ISGs, and independently of type I IFNs, alternative host cell factors interacting with bunyaviruses also have an antiviral action. The restriction factors currently known to exert an antiviral activity against bunyaviruses will be described hereafter, along with their mechanism of action, when it is known. Figure 2 represents these restriction factors as well as the step of the viral cycle which is inhibited.

### 6.1. Restriction Factors Targeting Bunyaviruses Entry

As mentioned before, bunyaviruses are enveloped viruses that enter the cells via endocytosis. Viral and cellular membrane fusion can occur in the early and late endosomes or lysosomes, depending on the virus. To date, several proteins have been described to interfere and inhibit the entry of bunyaviruses: the IFN-induced transmembrane (IFITM) proteins, the γ-IFN-inducible lysosomal thiol reductase (GILT) and the cholesterol-25-hydrolase (CH25H).

#### 6.1.1. IFN-Induced Transmembrane Proteins (IFITMs)

In humans, five IFITMs have been described (IFITM1, IFITM2, IFITM3, IFITM5 and IFITM10). They comprise two anti-parallel transmembrane domains, separated by a short cytoplasmic domain. They are basally expressed, but their expression is robustly induced by type I IFN. IFITM proteins have been widely studied for their roles in many biological processes, such as development and immune cell signaling [48]. Interestingly, an antiviral role of IFITM1/2/3 has been described against a broad range of viruses, such as influenza A virus (IAV) and human immunodeficiency virus type I (HIV-I) [49]. The mechanisms of IFITMs restriction activity are still not well characterized, but seem to be corroborated with their subcellular localization and appear to inhibit viral and cellular membrane fusion. Indeed, these transmembrane proteins are located mostly in early or late endosomes and lysosomes for IFITM2/3, whereas IFITM1 is located at the plasma membrane. Correspondingly, IFITM2/3 efficiently restrict viruses entering the cytoplasm via late endosomes, while IFITM1 more efficiently restricts viruses that enter the cytoplasm via direct fusion with the plasma membrane or with the membrane of early endosomes [49].

To date, IFITMs have been shown to inhibit the in vitro infection of several bunyaviruses from different families, including RVFV, LACV, Andes virus (ANDV) and HTNV [50,51] and Dugbe virus (DUGV) [52,53]. Some specificities exist: for example, only IFITM2/3 restrict RVFV, only IFITM1/3 restrict DUGV, while all three IFITMs inhibit LACV, ANDV and HTNV. For RVFV, a study suggested that IFITM2 and IFITM3 inhibit fusion between viral and endosomal membranes, and consequently the release of RNPs into the cytoplasm, as observed for other viruses such as IAV [50].

Although IFITM proteins inhibit some medically important bunyaviruses, its specificity remains to be understood. Indeed, IFITMs seem to have no effect on some other bunyaviruses, such as CCHFV [50]. A study using pseudoparticles expressing the envelope proteins of LCMV, LASV and MACV [54] revealed that they were not affected by IFITM expression, suggesting that all three viruses are refractory to IFITMs restricting activity, as CCHFV. Mechanisms of escape to IFITMs restricting activity have been described for other viruses. For instance, IAV suppresses the expression of IFITM3 by activating p53 or degrading eukaryotic translation initiation factor B (eIF4B) [55,56]. For CCHFV, LCMV, LASV and MACV, it remains to be understood how these viruses are able to escape IFITMs restriction.

#### 6.1.2. γ-IFN-Inducible Lysosomal Thiol Reductase (GILT)

The γ-IFN-inducible lysosomal thiol reductase (GILT) is a protein located in the lysosomes of dendritic cells, macrophages or B lymphocytes, which has a role in the degradation of cathepsin, as well as in facilitating antigen presentation by major histocompatibility complexes (MHC) I and II. Because of its lysosomal localization, its restriction activity is thus limited to viruses invading the cytoplasm via lysosomes, such as severe acute respiratory syndrome coronavirus (SARS-CoV), Ebola virus (EBOV), and from the *Bunyavirales* order, LASV, as shown by a study using pseudotyped retroviral particles expressing the envelope glycoproteins of these viruses [57]. The restriction activity of GILT is notably linked to its thiol reductase activity, as mutations of the reductase motif abolished GILT-mediated restriction of viral entry. Importantly, GILT reduces the proteolytic activity of cathepsin L, a lysosomal protease essential for the infectious entry of SARS-CoV and EBOV. GILT inhibition of cellular entry of SARS-CoV and EBOV could therefore be linked to the interference with cathepsin L metabolism in a thiol reductase-dependent manner. However, LASV entry does not rely on cathepsin L, explaining the necessity to further study the mechanism of action of GILT restriction activity against this virus.

#### 6.1.3. Cholesterol-25-hydrolase (CH25H)

The cholesterol-25-hydrolase (CH25H) and its enzymatic product 25-hydroxycholesterol (25HC) are important regulators of cholesterol homeostasis [58]. In brief, CH25H and 25HC inhibit the transcription factor sterol regulatory element-binding protein (SREBP) and activate the transcription factor liver X receptor (LXR). These two transcriptional factors have a great importance in modulating cellular cholesterol homeostasis. Moreover, 25HC regulates the location of cholesterol molecules in membranes. The CH25H gene is an ISG in mice and rhesus monkeys; yet whether CH25H is encoded by a human ISG is still debated [58].

Recently, CH25H and 25HC have been shown to exert an antiviral activity against a large number of highly pathogenic viruses, such as HIV-1 or EBOV. To date, two members among the *Bunyavirales* order are known to be inhibited by CH25H: RVFV [59] and LASV [60]. CH25H induces cellular membrane changes that impair viral entry at the step of membrane fusion for RVFV [59]. Of note, CCHFV entry is dependent on cholesterol [61], and CH25H could have an impact on this step, which has hitherto not been studied. For LASV, an alternative mechanism has been observed that differs from RVFV. Indeed, 25HC induces changes in the glycosylation of the GP1 glycoprotein of LASV. GP1 is still incorporated to newly formed virions, but its binding to α-dystroglycan, the LASV attachment and entry receptor, is impaired [60]. The mechanism by which 25HC induces these changes in GP1 glycosylation is yet to be defined.

### 6.2. Restriction Factors Interfering with Genomic Transcription, Protein Translation and Genomic Replication

Following the entry steps, the viral genome is released into the cell cytoplasm and a first step of transcription occurs, followed by a step of replication. Numerous host factors are known to interfere with these steps, as described in this review.

#### 6.2.1. Myxovirus Resistance Protein A (MxA)

The Mx family GTPases is composed of MxA and MxB in humans (and Mx1 and Mx2 in rodents). These type I and type III IFN-induced proteins have a well-described antiviral activity against a broad range of viruses, with both RNA and DNA viruses being susceptible to their antiviral action. They have a large GTPase domain at their amino-terminus, followed by a central interacting domain and a carboxy-terminal leucine zipper domain, and are located near the smooth endoplasmic reticulum [62].

The antiviral action of MxA against LACV is the most extensively described in the *Bunyavirales* order, both in vitro and in vivo [39,63,64,65,66,67]. Its potential mechanism of action relies on its interaction with LACV NP to sequester it in perinuclear complexes, rendering it unavailable for the encapsidation of viral RNA, and thus interfering with genome replication [64,65]. CCHFV and DUGV are also inhibited in vitro by MxA [68,69]. For CCHFV, as seen for LACV, MxA induces a perinuclear re-localization of CCHFV NP. However, this antiviral activity of MxA was not confirmed in vivo for DUGV. Indeed, transgenic *Ifnar*^−/−^ mice constitutively expressing MxA were as sensitive to DUGV infection as *Ifnar*^−/−^ mice, suggesting that the presence of MxA alone does not confer any protection [38]. For hantaviruses, the role of MxA is also controversial. MxA gene expression is upregulated during infection with hantaviruses [70,71,72]. While some studies revealed an inhibition of genome replication for HTNV, Puumala virus (PUUV) and Tula virus (TULV) when MxA is overexpressed [63,73], another study showed that *MxA* silencing by knock-down (KD) did not impair the IFN-induced inhibition of HTNV [74]. Finally, two members of the *Phenuiviridae* family, RVFV and sandfly fever Naples virus (SFNV), are also both inhibited upon MxA overexpression [63,67,75], and a study relying on the formation of RVFV virus-like particles (VLPs) demonstrated that the viral primary transcription is the cycle step targeted by MxA [76].

#### 6.2.2. Protein Kinase R (PKR)

The protein kinase R (PKR) is a serine-threonine kinase that is constitutively expressed, albeit in a latent and inactive form. Its expression is upregulated by type I IFNs. To be activated, it needs to encounter, notably, dsRNA or 5′ppp ssRNA. Upon binding to one of these triggers, PKR auto-phosphorylates and dimerizes. Once dimerized, active PKR phosphorylates the α-subunit of the eukaryotic translation initiation factor eIF2 (eIF2α), which stops the translation process [62]. Because viruses need the cellular translation machinery to translate their proteins, PKR is considered as a restricting factor for a high number of viruses, including BUNV [77,78]. Indeed, BUNV infection leads to PKR-dependent phosphorylation of eIF2α and in vivo studies performed on PKR-deficient laboratory mice revealed a higher susceptibility to BUNV infection compared to wild-type mice [78]. As for RVFV, a natural isolate with a large depletion in the NSs gene (Clone 13) is susceptible to PKR restriction, both in vitro and in vivo [79,80]. Compared to Clone 13, RVFV expressing a complete NSs is much less susceptible to PKR restriction. These results suggest an antagonism function of RVFV NSs protein against PKR-mediated viral restriction, which is later discussed in this review.

However, PKR does not restrict all bunyaviruses. Indeed, infection of PKR-deficient laboratory mice with DUGV did not have an impact on their survival rate, suggesting that PKR alone does not restrict the replication of this virus [38]. Moreover, while PKR was activated in human cells upon infection by some arenaviruses (more precisely JUNV and MACV), it did surprisingly not affect their viral replication. In the same study, LASV infection of human cells did not activate PKR [81].

#### 6.2.3. IFN-Induced Protein with Tetratricopeptide Repeats 1 (IFIT1)

The IFN-induced protein with tetratricopeptide repeats 1 (IFIT1) is a protein whose ability to bind the 5′ppp end of RNA was discovered by co-immunoprecipitation of agarose beads coupled to 5′ppp RNA in uninfected human embryonic kidney cells (HEK293) followed by mass spectrometry experiments [82]. Following this discovery, the authors performed KD experiment by small interfering (si) RNA of IFIT1 in HeLa cells and assessed its effect in a context of infection. This KD led to an increased replication of NSs-deficient RVFV Clone 13, suggesting that IFIT1 is a restriction factor against RVFV [82]. The mechanism of action of this restricting activity might be linked to IFIT1 binding to 5′ppp RNA, but this remains to be confirmed.

#### 6.2.4. Long Isoform of Mono-ADP-ribosyltransferase 12 (PARP12L)

The poly (ADP-ribose) polymerases (PARPs) are enzymes catalyzing the transfer of ADP-ribose units to target proteins using a nicotinamide adenine dinucleotide (NAD^+^) as substrate. More specifically, PARP12 is a mono-ADP-ribosyltransferase whose expression is IFN-induced and whose long isoform, PARP12L, was discovered to have an antiviral effect against a virus from the *Alphaviridae* family, namely Venezuelan equine encephalitis virus (VEEV). This antiviral activity was found to be broad-spectrum, since the replication of RVFV was also inhibited by PARP12L in an overexpression system of PARP12L [83]. For VEEV, it was shown that PARP12L inhibits cellular and viral translation, like PKR. However, it appears that PARP12L uses a different mechanism than PKR to inhibit translation, involving its binding to polysomes [84]. The same mechanism for RVFV PARP12L restricting activity could be proposed but needs confirmation.

#### 6.2.5. Decapping Protein 2 (Dcp2)

Decapping 2 (Dcp2) is a protein located in the P-bodies which is involved in the mRNA decapping machinery. This machinery is essential to ensure the cellular RNA turnover, by removing the protecting 7-methylguanylate (7 mG) cap present at the 5′ end of cellular mRNAs. mRNAs ripped of their 5′ cap expose a 5′ monophosphate, which is target for cellular exonucleases, thus begetting RNA degradation.

A genome-wide RNA interference (RNAi) screen performed in *Drosophila* cells, which are used as an insect cell model useful to study arboviruses interactions with their vector, identified Dcp2 as a potential host restriction factor for RVFV. Depletion or overexpression experiments of Dcp2 in *Drosophila* cells led respectively to an increase and decrease of RVFV transcription. These results were confirmed in a RNAi performed in adult flies: Dcp2-depleted flies succumbed to RVFV infection, whereas control flies presented little mortality. These results were extended to another bunyavirus transmitted by mosquitoes, LACV [85]. A similar finding was described in plant cells for the tomato spotted wilt virus (TSWV), a plant bunyavirus from the *Tospoviridae* family [86]. Notably, bunyaviruses have developed a cap-snatching mechanism in order to protect their mRNAs and initiate their translation [18,87]. Indeed, several strategies have been developed by viruses to ensure the stability of their mRNA. All rely on the protection of their 5′ ends from cellular exonucleases [88], whether it is by encoding their own capping machinery, by attaching a small, viral-encoded protein to the 5′ end, or by stealing the 5′ end of host mRNAs thanks to a viral-encoded endonuclease. This last strategy, called cap-snatching, ensures that viral mRNAs become indistinguishable from endogenous mRNAs. Thus, it is thought that a competition between the viral endonuclease responsible for cap-snatching and Dcp2 for cellular mRNA caps is at the origin of the restricting activity of Dcp2. However, these results remain to be confirmed in mammalian cells.

#### 6.2.6. DDX17

DDX17 is a DEAD-box RNA helicase that has a major role in RNA metabolism. DDX17 is the human homolog of Rm62, whose role in antiviral defense against both RVFV and LACV was identified through RNAi screens in *Drosophila cells* and in adult flies [89]. Conservation of the antiviral role of Rm62 in human cells was observed, as *DDX17* silencing in human cells led to an increased viral replication of both RVFV and LACV. More precisely, DDX17 can interact with RVFV RNA, likely via the recognition of defined structured viral RNA elements, notably the hairpin-like structures present in the intergenic region on the S segment (between N and NSs) and in the 5′ non-coding region of the S segment. It remains to be understood how this binding mediates an antiviral activity, but it can be hypothesized that DDX17 acts as a sensor protein bringing viral RNA to the degradation machinery, as it is known to interact with exonucleases or proteins involved in the decapping machinery, like Dcp2. As a helicase, DDX17 may also unwind viral RNA to facilitate its degradation. A recent biophysical study obtained evidence in support of the latter hypothesis. Indeed, it was observed in vitro that DDX17 helicase domain interacts with and unwinds the intergenic region of RVFV S segment along with S 5′ non-coding region [90].

#### 6.2.7. IFN-Induced Protein 44 (IFI44)

Little is known about the IFN-induced protein 44 (IFI44, previously known as MTAP44). It was initially discovered in the context of hepatitis C virus (HCV) infection, for which it does not have any antiviral activity described. Interestingly, its overexpression leads to BUNV restriction in vitro, but the involved mechanism is yet to be understood [77]. However, in another study, IFI44 silencing by siRNA negatively affected LCMV replication, suggesting that IFI44 is not a host restriction factor, but rather a host dependency factor for LCMV [91].

#### 6.2.8. Promyelocytic Leukemia Protein (PML)

Promyelocytic leukemia protein (PML) nuclear bodies (NBs) are nuclear structures containing several proteins, among which PML, that contribute to the regulation of a large number of key cellular processes, such as cell cycle progression, DNA damage response, transcriptional regulation and apoptosis. PML expression is induced by type I IFNs.

In a context of LCMV infection, it was shown that PML-deficiency in murine embryonic fibroblasts leads to an increased LCMV replication and that overexpression of PML results in diminished LCMV replication [92]. This LCMV-directed antiviral activity of PML was confirmed in vivo in PML-deficient LCMV-infected laboratory mice, which exhibited an accelerated onset of symptoms and higher viral loads compared to control mice [93]. Upon LCMV infection, it was shown that PML NBs are relocated to the cytoplasm following an interaction of PML with the arenaviral Z protein [94]. However, the consequences of this re-localization as well as the exact mechanism by which PML interferes with LCMV replication remain to be understood.

#### 6.2.9. Moloney Leukemia Virus 10 Protein (MOV10)

Moloney leukemia virus 10 protein (MOV10) is an RNA helicase with an important role in regulating cellular mRNA stability and translation. Moreover, it has an antiviral activity against a high number of viruses, including retroviruses [95], hepatitis B virus (HBV) [96], HCV [97] and some RNA viruses (vesicular stomatitis virus (VSV) [98] and IAV [99]). For retroviruses like HIV-1, MOV10 is known to interfere with viral reverse transcription, which could explain its antiviral activity. However, for some other viruses, like hepatitis D virus, MOV10 has a proviral rather than an antiviral activity [100].

The role of MOV10 during bunyaviral infections was only recently studied [101]. It was shown that SFTSV infection specifically upregulates MOV10 expression in vitro and that MOV10 inhibits the replication of SFTSV and other related viruses (Heartland virus (HRTV) and Guertu virus (GTV)) in infected cells. This antiviral activity of MOV10 was confirmed in vivo in *MOV10*-KD mice, in which SFTSV viral loads were significantly lower than in control, SFTSV-infected mice. The helicase function of MOV10 was found to be dispensable for MOV10 SFTSV-directed antiviral activity. However, MOV10 interacts with the NP of SFTSV, and this interaction was found to be essential for MOV10 antiviral activity, as it impairs NP oligomerization and NP interactions with viral RNA and with the L protein. Thus, the assembly of RNPs is inhibited. As the NP of SFTSV shows only low sequence identity with other bunyaviruses, it remains to be further investigated whether MOV10 has an antiviral activity against other members of the order.

#### 6.2.10. IFN-Stimulated Gene 20 (ISG20)

IFN-stimulated gene 20 (ISG20) is an exonuclease known as a restriction factor for multiple viruses, including for instance HBV and HCV [102,103]. In a flow-cytometry-based gain-of-function screen of 488 ISGs, ISG20 was notably found to be a potent restriction factor against BUNV [53]. As seen for HBV, ISG20 antiviral activity against BUNV is linked to its exonuclease activity. Indeed, mutations in its active exonuclease site abrogated its antiviral effects against BUNV. The antiviral effect against BUNV of endogenous ISG20 was confirmed by short hairpin (sh) RNA-mediated KD of *ISG20*. Moreover, in vitro gain-of-function experiments conducted upon other bunyaviral infections demonstrated a specific effect of ISG20. Indeed, while DUGV and PUUV also were inhibited by the overexpressed ISG20, phenuiviruses appeared resistant to ISG20 antiviral activity.

#### 6.2.11. 2′-5′-Oligoadenylate Synthetase (2′-5′-OAS)/RNase L

The 2′-5′-oligoadenylate synthetase (2′-5′-OAS) is a constitutively expressed enzyme whose production is upregulated by IFN and which is activated by dsRNA. It catalyzes the synthesis of 2′-5′-oligoadenylates, which bind and activate the latent endoribonuclease RNase L. Once activated, RNase L degrades ssRNAs and therefore inhibits viral replication [62]. As such, it is considered as a potent restriction factor for a broad range of viruses. Concerning bunyaviruses, only a few studies have attempted to determine the implication of 2′-5′-OAS/RNase L system as a host restriction factor. Some microarray experiments performed on cells infected with hantaviruses revealed an upregulation of the expression of the 2′-5′-OAS [70,71], however further studies are needed to determine the consequences of this upregulation. Moreover, the depletion of 2′-5′-OAS by RNAi led to an increase in the viral replication of a NSs-depleted RVFV, suggesting a potential restricting role of 2′-5′-OAS that still needs to be characterized [104]. However, the 2′-5′-OAS/RNase L system does not seem to restrict BUNV replication, as shown by in vivo experiments conducted in RNase L-deficient mice [78].

The 2′-5′-oligoadenylate synthetase (2′-5′-OAS) is a constitutively expressed enzyme whose production is upregulated by IFN and which is activated by dsRNA. It catalyzes the synthesis of 2′-5′-oligoadenylates, which bind and activate the latent endoribonuclease RNase L. Once activated, RNase L degrades ssRNAs and therefore inhibits viral replication [62]. As such, it is considered as a potent restriction factor for a broad range of viruses. Concerning bunyaviruses, only a few studies have attempted to determine the implication of 2′-5′-OAS/RNase L system as a host restriction factor. Some microarray experiments performed on cells infected with hantaviruses revealed an upregulation of the expression of the 2′-5′-OAS [70,71], however further studies are needed to determine the consequences of this upregulation. Moreover, the depletion of 2′-5′-OAS by RNAi led to an increase in the viral replication of a NSs-depleted RVFV, suggesting a potential restricting role of 2′-5′-OAS that still needs to be characterized [104]. However, the 2′-5′-OAS/RNase L system does not seem to restrict BUNV replication, as shown by in vivo experiments conducted in RNase L-deficient mice [78].

### 6.3. Restrictions Factors Blocking Viral Assembly and Egress

The last steps of the viral cycle lead to the clustering of virus assembly factors at the site of assembly, the assembly and envelopment of viral particles, and the egress of newly formed virions. Relative to other viral cycle steps, only few restriction factors have been described to play a role at this stage. Among them, viperin (virus inhibitory protein, endoplasmic reticulum-associated, IFN-inducible) and tetherin have both been identified as restriction factors of some viruses from the *Bunyavirales* order.

#### 6.3.1. Viperin

Viperin is encoded by an ISG and localizes in the endoplasmic reticulum and in lipid droplets. Its antiviral action has been described for a large number of viruses.

Notably, viperin is upregulated upon LCMV infection [105]. Similar findings were reported for JUNV, and viperin overexpression led to a strong antiviral effect against JUNV by inhibiting infectious virus release [106]. This phenomenon may be explained by two mechanisms. First, viperin overexpression leads to a mislocalization of viral glycoproteins, hence perturbing viral assembly or virion morphogenesis. Second, it leads to an altered lipogenesis that could prevent JUNV to use the lipid rafts for virion budding. However, viperin could also have an antiviral role at an earlier step of JUNV cycle. Indeed, it has been shown that viperin overexpression in JUNV-infected cells leads to a decreased viral RNA transcription. Moreover, JUNV NP interacts with viperin. Taken together, these data suggest that viperin might interact with JUNV NP to counteract its function on genome viral transcription and replication [106].

BUNV has also been shown to be regulated by viperin [77]. However, unlike JUNV, viperin expression is not upregulated upon BUNV infection, except when a recombinant virus whose NSs has been deleted is used, suggesting a role of NSs to counteract the regulatory action of viperin. The exact mechanism of viperin restriction activity for BUNV remains to be elucidated.

#### 6.3.2. Tetherin

Tetherin, also known as bone marrow stromal antigen 2 (BST-2), is encoded by an ISG. This protein is a type II transmembrane protein whose ectodomain structure is linked to an amino-terminal transmembrane region and a glycosylphosphatidylinositol (GPI) anchor at its carboxy-terminus. In humans, tetherin is located at the *trans-*Golgi network (TGN), endosomes and within lipid rafts at the plasma membrane.

Tetherin was initially found to be a potent antiviral factor of HIV-1 that is counteracted by the viral protein Vpu. Its name reflects its ability to restrict the release of HIV-1 by retaining the viral particle at the cell membrane, like an anchor. This protein has a propension to form homodimers, and it was also shown that it can be incorporated into the viral membrane of HIV-1 [107]. Therefore, a model of antiviral activity has been proposed for this protein in which homodimerization between tetherin monomers from cellular and viral membranes is at the origin of the retention of the virion at the cell membrane.

Since then, tetherin was found to inhibit a large number of enveloped viruses [108], including bunyaviruses such as arenaviruses like LASV, MACV and JUNV [109]. Notably, the release of LASV and JUNV VLPs based on the expression of its matrix protein Z [110] as well as infectious LASV, JUNV and MACV [109,111] is restricted by tetherin. The mechanism does not involve tetherin dimerization [112], in contrast to what had first been proposed for HIV-1. Moreover, further studies are needed to know whether tetherin is incorporated into the membrane of arenaviruses, as seen for HIV-1. Notably, RVFV was found to be refractory to tetherin restriction activity [109].

Tetherin demonstrates species-related specificities, which have repercussions on its ability to inhibit some viruses from the *Bunyavirales* order, such as peribunyaviruses. This family contains viruses which are pathogenic for humans (like OROV and LACV), for ruminants (like SBV, Akabane virus (AKAV) and Sathuperi virus (SATV)), or both (like Cache Valley virus (CVV)). Interestingly, SBV, AKAV and SATV (which are viruses with a ruminant tropism) are restricted by human tetherin, but not by sheep tetherin. For SBV, human tetherin was found to induce a reduction of the incorporation of the Gc glycoprotein into SBV virions. Conversely, OROV and LACV (which are viruses with a human tropism) are restricted by sheep tetherin, but not by human tetherin. CVV, which has both a human and a ruminant tropism, was restricted by neither human tetherin nor sheep tetherin. Thus, the species-related specificities of tetherin might in part explain the host species range of these pathogens [113].

## 7. Escape of Antiviral Responses by Bunyaviruses

**Figure 3:**
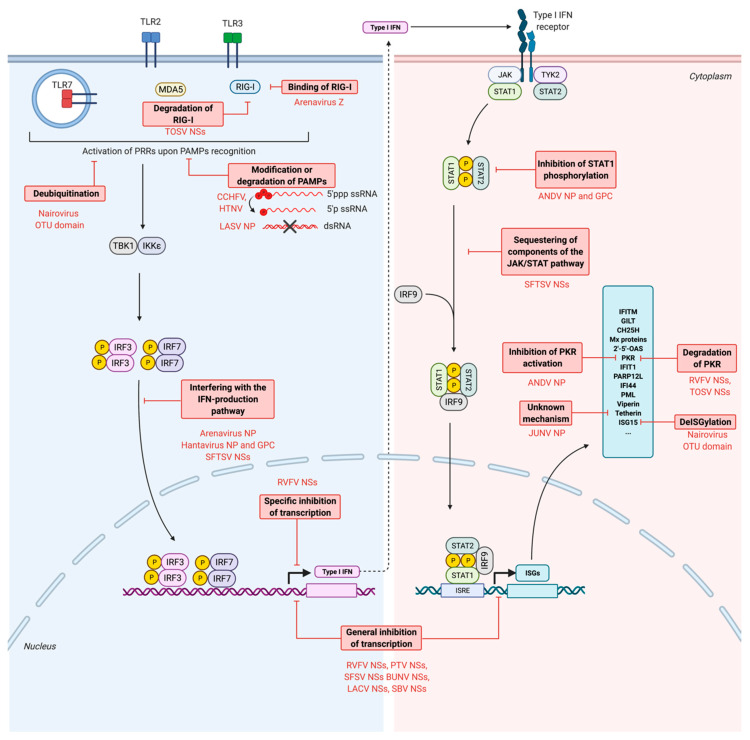
Viral strategies counteracting the host intrinsic and innate immunity system. Upon detection of pathogen-associated molecular patterns (PAMPs) by pattern recognition receptors (PRRs), pathways are activated leading to the production of type I interferon (IFN). One of them involves the phosphorylation, dimerization and nuclear translocation of IFN regulatory factors 3 and 7 (IRF3 and IRF7). Type I IFN are secreted and act in an autocrine and paracrine manner. Upon recognition by the type I IFN receptor, the signal transducer and activator of transcription proteins 1 and 2 (STAT1 and STAT2) are phosphorylated and associate as a heterodimer, as well as with the IFN regulatory factor 9 (IRF9). This complex translocates to the nucleus to activate the transcription of IFN-stimulated genes (ISGs). Viral countermeasures developed by viruses to inhibit these pathways at different steps are written in red boxes. GPC: glycoproteins precursor; NP: nucleoprotein; OTU domain: ovarian tumor domain. IKKε: IκB kinase-ε; IRF: IFN regulatory factor; ISG: IFN-stimulated gene; ISRE: IFN-stimulated response element; JAK: janus kinase; MDA5: melanoma differentiation-associated protein; RIG-I: retinoic acid-inducible gene-I; STAT: signal transducer and activator of transcription; TBK1: TANK-binding kinase 1; TLR: Toll-like receptor; TYK2: tyrosine kinase 2. ANDV: Andes virus; CCHFV: Crimean-Congo hemorrhagic fever virus; JUNV: Junín virus; HTNV: Hantaan virus; LACV: La Crosse virus; LASV: Lassa virus; LCMV: lymphocytic choriomeningitis virus; PTV: Punta Toro virus; RVFV: Rift valley fever virus; SBV: Schmallenberg virus; SFTSV: severe fever with thrombocytopenia virus; SFSV: Sandfly fever Sicilian virus; TOSV: Toscana virus.

Bunyaviruses have developed numerous strategies to escape the antiviral responses. As seen previously, a large number of known bunyaviruses restricting factors are dependent on the IFN response. Thus, to escape efficiently host antiviral responses, bunyaviruses either inhibit the production of IFN or diminish the IFN-induced response. But some restricting factors also are counteracted directly by bunyaviruses, such as PKR and tetherin. The different strategies developed to escape host antiviral responses are schematically summarized in Figure 3.

### 7.1. Escape from the PRRs Recognition

In order to inhibit the production of IFN which leads to an antiviral state, some viruses act at the very beginning of the IFN production signaling cascade. They avoid being recognized by the PRRs through strategies involving the modification or degradation of PAMPs.

#### 7.1.1. Modification of PAMPs: Processing of the Genome

Bunyaviruses do not produce high amounts of dsRNA, which is an important PAMP [114]. However, their ssRNA genome possesses a 5′ppp end, which is a potent trigger of RIG-I-mediated IFN production [24]. Yet, as already described, both nairoviruses and hantaviruses have developed an alternative strategy that is supposed to avoid recognition by RIG-I: during replication, the 5′ end of their genome is processed to be mono-phosphorylated rather than tri-phosphorylated [24]. The exact mechanism by which these 5′p ends are generated remains to be fully understood. However, despite this strategy, both CCHFV and HTNV still induce a RIG-I-dependent IFN production [26,27], suggesting that an alternative PAMP is at the origin of a RIG-I-mediated detection of viral RNAs by the host cell. The role of HTNV NP in RIG-I activation was already mentioned in this review [26]. Moreover, the complementary antigenomic RNA could be a PAMP itself, as it does interact with less affinity than genomic RNA with the NP, as shown for hantaviruses [115], and could thus be more accessible to RIG-I sensing by lack of NP-mediated protection from detection. Alternatively, the production of 5′ monophosphorylated ends might involve the generation of 5′ppp intermediates or truncated defective interfering (DI) particles, which could be putative PAMPs. More studies are necessary to determine the PAMPs at the origin of the PRR-mediated IFN production.

#### 7.1.2. Degradation of PAMPs

dsRNA is an important PAMP and is recognized by RIG-I and PKR. Even though bunyaviruses do not produce high amounts of dsRNA, it can still trigger the activation of these sensors. To inhibit the dsRNA-mediated activation of RIG-I and PKR, LASV NP exhibits a dsRNA-specific exonuclease activity. This activity was shown to inhibit the accumulation of viral PAMPs, and consequently enables the virus to escape PRR recognition [116,117,118,119].

### 7.2. Inhibition of the Production of IFNs

Inhibiting the production of IFNs is an important and widely developed strategy by viruses to avoid the establishment of an antiviral state. Bunyaviruses inhibit the production of IFNs by either inducing a cellular transcriptional and translational shutoff or interfering with the signaling cascade leading to the production of IFN.

#### 7.2.1. Global Cellular Transcriptional and Translational Shutoff

The S segment of bunyaviruses, with the exception of arenaviruses, encodes a non-structural protein termed NSs, which has already been mentioned as a modulator of several restriction factors. The NSs protein of RVFV was the first one to be identified as an IFN-antagonist [35,120]. Since then, the NSs proteins from other phenuiviruses (PTV [121], Toscana virus [122], Sandfly fever Sicilian virus (SFSV) [123], SFTSV [124]), from peribunyaviruses (LACV [37], BUNV [125,126], OROV [127], SBV [128]), or from hantaviruses (TULV and PUUV [129], ANDV [130]) also were found to be IFN-antagonists.

RVFV NSs counteraction of the IFN-mediated response is the most extensively studied and relies on its ability to induce a global cellular transcriptional shutoff [131]. Indeed, it was shown that NSs interacts with the p44 component of the transcription factor II H (TFIIH), a general transcription factor that recruits the RNA polymerase II to the promoters, and sequesters it into nuclear filamentous structures to prevent the assembly of TFIIH [132]. Moreover, it also interacts with the p62 subunit of TFIIH [133] and promotes its proteasomal degradation [134] by recruiting the F-box protein FBXO3 [135], a substrate recognition component of modular E3 ubiquitin ligases of the Skp1, Cullin1, F-box (SCF) protein type. Finally, RVFV NSs expression was also reported to correlate with a block of nuclear host mRNA export [136]. As a result of these events, IFN production is negatively impacted. However, NSs also specifically targets IFN synthesis by interacting with a component of a host transcriptional suppressor complex specifically regulating IFN-ß expression, the Sin3A-associated protein (SAP30). Indeed, a recombinant RVFV virus in which NSs is deleted for the region of interaction with SAP30 was found to be unable to inhibit IFN response and to be avirulent in mice [137]. The interaction of NSs with SAP30 leads to the formation of a repression complex on the IFN-ß promoter. Consequently, the IFN-ß production is negatively impacted.

Some differences in NSs functions are found among phenuiviruses. For example, as RVFV NSs, PTV and SFSV NSs inhibit host transcription. On the contrary, TOSV NSs does not affect cellular transcription [122]. Finally, UUKV NSs was found to only have a weak IFN-antagonist effect [138].

Notably, phenuiviruses NSs is expressed in an ambisense coding strategy from the S segment. Thus, it is expected to be expressed only at later stages of infection, during the secondary transcription round. This is contradictory with its IFN-antagonism activity, which happens at early stages of infection. However, it was reported that antigenomic RNA segments, like genomic segments, are packaged into the virions for both RVFV and UUKV and are therefore transcribed during primary transcription [139,140,141]. Thus, NSs protein is also likely produced at early stages of infection.

As for peribunyaviruses, NSs induces a massive and rapid inhibition of cellular mRNA transcription. Indeed, NSs of both BUNV and LACV directly interfere with the cellular RNA polymerase II, by reducing the phosphorylation of its carboxy-terminal domain for BUNV (which is necessary for mRNA elongation) [126] and by additionally degrading its large subunit RPB1 for LACV by a mechanism involving the cellular E3 ubiquitin ligase subunit, Elongin C [142,143]. Like LACV, SBV NSs also induces RPB1 degradation [128,144].

CCHFV was also reported to express a NSs protein that undergoes rapid proteasomal degradation [15]. However, an IFN-antagonist activity was not described for this protein. To date, whether other members of the *Nairoviridae* family encode a functional NSs is unknown. It is therefore expected that nairoviruses have developed an NSs-independent strategy to escape the innate immune response, as mentioned hereafter.

#### 7.2.2. Interference with the IFN Production Pathway

- Alteration of post-translational modifications of key factors from the IFN production pathway: deubiquitination and deISGylation

The type I IFN response is highly regulated and relies, notably, on post-translational modifications of several key actors of the type I IFN response by ubiquitin (Ub) [145,146]. For instance, K63-linked polyubiquitination is necessary for the activation of several components of the pathway, including RIG-I [147]. Global ubiquitination is regulated by a number of cellular deubiquitinases (DUB), among which the ovarian tumor protease (OTU) subfamily [148,149].

Relative to other bunyaviruses, nairoviruses encode a particularly large L protein of roughly 12 kb. Studies showed that several nairoviruses have an OTU-like protease motif at the N-terminus of their L protein [150,151,152], which is absent in the L proteins of other bunyaviruses, except for one plant virus from the *Phenuiviridae* family, the rice stripe tenuivirus (RSV), which also encodes an OTU domain [153,154]. The similarity of this domain with cellular OTU proteins suggests that it may be involved in deubiquitination. Indeed, several biochemical and structural analysis of OTU domains of different nairoviruses corroborate this hypothesis [155,156,157,158,159]. Biochemical and structural studies of nairoviral OTU domains first focused on CCHFV or closely CCHFV-related nairoviruses, including NSDV, Ganjam virus (GANV), DUGV and Erve virus (ERVEV) [160,161,162,163,164]. However, recently, two global studies comparing the DUB activity of 12 nairoviruses [165] showed that nairoviral OTUs differ in their DUB activity and can therefore be divided into groups possessing high, moderate or low deubiquitinase activity. These differences can notably be explained by the fact that there is a high degree of diversity between nairoviral OTUs, with sometimes less than 25% amino acid identity with CCHFV OTU domain [165]. However, globally, a higher activity toward K6-, K11-, K48- and K63-linked Ub was observed. By disrupting these ubiquitinase functions, nairoviral OTUs dampen antiviral responses.

**Figure 4:**
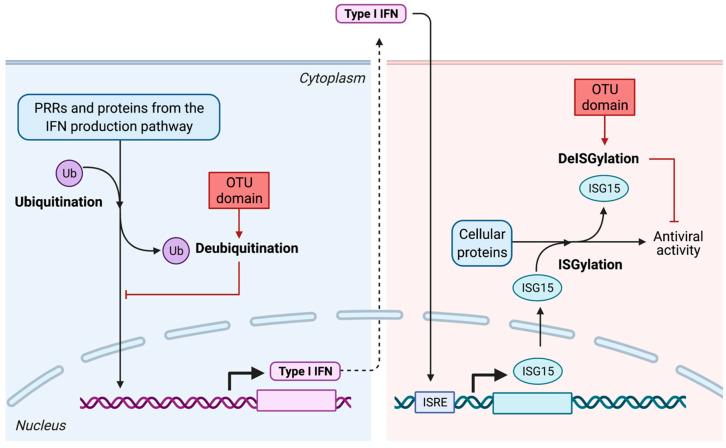
Deubiquitination and deISGylation activity of the nairovirus OTU domain. IFN: interferon; ISG15: IFN-stimulated gene 15; ISRE: IFN-stimulated response element; OTU domain: ovarian tumor domain; Ub: ubiquitin.

By removing post-translational modifications essential for the innate immune signaling pathways, the nairoviral OTU domains are able to dampen antiviral responses, as shown by several studies relying on the overexpression of the isolated OTU domains [155,156,157] (Figure 4). Moreover, the immunomodulatory effects of DUB activities of the CCHFV OTU domain have been observed in a reverse genetic system. Indeed, a reduced activation of key components of the innate immune response (including RIG-I) was observed upon infection with wild-type CCHFV, while infection with a mutated CCHFV whose OTU lacked DUB activity led to the establishment of an antiviral state in infected cells [159]. However, when associated with the full-length L protein, the ability of the OTU domain to suppress the type I IFN-mediated immune response seems to be reduced [155,156,157] and a study using a CCHFV VLP system with a protease-negative OTU mutant in immunocompetent cells revealed that IFN induction was not impacted upon infection [166], adding more complexity to the understanding of the contribution of the OTU domain in the antiviral response.

It is worth noting that studies on nairoviral OTU domains also highlighted an additional activity of viral OTUs compared to cellular DUB proteins of the OTU family, namely the deISGylase activity [155,156,157,158,159] (Figure 4). Type I IFN induces the expression of a 15 kDa protein, the IFN-stimulated gene 15 (ISG15), which is a member of the ubiquitin family [167]. ISG15 can be covalently conjugated onto target proteins via an enzymatic cascade called ISGylation [168]. This protein, conjugated or in its free intracellular or extracellular form, is known to antagonize viral replication during acute and latent infections, although its activity, being diverse, pathogen- and host species-dependent [169,170], is not well understood. Interfering with ISG15 conjugation could enable nairoviruses to escape the innate immune response. As for the DUB activity of nairoviral OTUs, the deISGylase activity of nairoviral OTUs also is diverse among nairoviruses, which could reflect an adaptation of the virus to its host. Indeed, ISG15 is a protein that is found only in vertebrates and that is highly divergent between species. The preferences toward the ISG15 from one or another species generally reflects the preferential hosts infected by the virus [171], hinting that a co-evolution process may have contributed to these specificities.

- Inhibition of the activation of RIG-I

RIG-I is one of the main sensors of bunyaviruses infection at the origin of IFN production. Some viruses from this order have therefore developed strategies to inhibit its activation. For instance, TOSV NSs interacts with RIG-I and triggers its proteasomal degradation [172].

As for arenaviruses, transfection of LCMV NP and LCMV RNA led to a decrease in LCMV RNA-induced activation of the type I IFN in vitro [28]. Moreover, LCMV NP was found to bind both RIG-I and MDA5. Yet, two NP mutants that do not inhibit the type I IFN signaling pathway also bind both RLRs, suggesting that RIG-I or MDA5 binding by NP is not sufficient to inhibit their activation and suggesting the necessity of another viral factor to efficiently counteract the RLR-mediated antiviral effects for these viruses [28]. In fact, the Z protein encoded by the L segment of arenaviruses was reported to inhibit the activation of RIG-I. Indeed, the Z proteins of four New-World arenaviruses, Guanarito virus (GTOV), JUNV, MACV and Sabiá virus (SBAV) were reported to bind RIG-I by co-immunoprecipitation experiments, which resulted in the downregulation of the IFN response [173]. However, in this study, this property was not shared by Old-World arenaviruses like LCMV or LASV, whereas in another study, all pathogenic arenaviruses, including LCMV and LASV, were found to have a Z-mediated inhibition of RLR-induced IFN production [174]. These controversial results can be explained by differences in the experimental systems, such as for instance the Z expression levels. The study of Z proteins in an infectious context is therefore necessary to better understand whether they are able to counteract the innate immune responses.

- Interference with the IFN production pathway

Another strategy for viruses to escape the host innate immunity is to inhibit the production of IFN by disrupting the signaling cascade leading to its production. Bunyaviruses have developed several ways to achieve this.

For instance, the arenaviral NPs inhibit the nuclear translocation and the transcriptional activity of IRF3 and of the nuclear factor κ-light-chain-enhancer of activated B cells (NF-κB), both key factors of the IFN production pathway [28,175,176,177]. More specifically, LCMV and JUNV NPs associate with IKKε and block its ability to phosphorylate IRF3 [178].

Hantaviruses NPs are also known to antagonize type I IFN responses [179,180,181]. More precisely, HTNV NP reduces the nuclear translocation of NF-κB, by interacting with importin α proteins involved in the nuclear shuttling of NF-κB, thus inducing a sequestration of NF-κB into the cytoplasm and inhibiting IFN production [179,180,181,182]. However, the NP proteins of other hantaviruses (ANDV, SNV, New York virus (NYV) and PHV) have no effect on NF-κB activation [183]. But recent studies revealed that ANDV NP is able to inhibit TBK1 and IKKε activation by an as yet unknown mechanism [183,184,185]. This activity of ANDV NP seemed not to be shared by other viruses, like SNV, NYV and PHV. Additionally, ANDV GPC was also found to inhibit the type I IFN induction, as well as SNV GPC [185]. Moreover, several reports showed that the ectopic expression of the cytoplasmic tail of the glycoprotein Gn of some hantaviruses (ANDV, NYV and TULV) leads to the inhibition of IRF3 activation [186,187,188]. This shows that glycoproteins can also be involved in the antagonism of the cellular antiviral responses by blocking IFN production. Furthermore, a study focusing on ANDV NSs protein showed that it acts as an IFN-antagonist by suppressing signals downstream of the RIG-I/MDA5 pathway but upstream of TBK1 [130].

Like the NSs of other phenuiviruses, SFTSV NSs has an immunomodulatory role, but its mechanism is different than the other phenuiviruses NSs. Indeed, SFTSV NSs is concentrated in cytoplasmic structures where it interacts with and sequesters several components of the RIG-I pathway, like RIG-I itself, tripartite motif-containing 25 protein (TRIM25) (a RIG-I-activating E3 ubiquitin ligase), TBK1 and IRF7 [23,124,189,190,191,192,193,194]. The IFN production pathway is therefore disrupted.

### 7.3. Inhibition of the Production of ISGs

Another strategy developed by viruses to inhibit the antiviral effects of IFN is to inhibit the production of ISGs, by interfering with the JAK/STAT pathway. For ANDV, this mechanism relies on the NP- and GPC-induced inhibition of STAT1 phosphorylation and nuclear translocation, thus inhibiting the production of ISGs [185,195]. For SNV, another hantavirus, while NP was not able to inhibit the IFN-induced transcription of ISGs, GPC was [185].

NSs of SFTSV was already mentioned for its ability to induce the sequestration of key components of the IFN production pathway. In addition, it was also found to sequester STAT1 and STAT2 into inclusion bodies, therefore interfering with the production of ISGs [196].

### 7.4. Inhibition of the Activity of Restriction Factors

Finally, some aforementioned restriction factors are directly inhibited by bunyaviral components and are consequently unable to exert their restricting activity.

#### 7.4.1. Inhibition of PKR

As previously mentioned, RVFV NSs has an important role in inhibiting IFN production by inducing a global as well as an IFN-specific transcriptional shutoff. However, IFN is still produced during RVFV infection and leads to the expression and activation of ISGs, like PKR. RVFV NSs notably counteracts the aforementioned PKR-induced inhibition of translation by inducing its proteasomal degradation [79,80,131]. This degradation is initiated by the recruitment by NSs of two F-box proteins, FBXW11 and β-TRCP1 [197,198]. Once more, differences exist between the NSs of phenuiviruses. Indeed, TOSV NSs efficiently induces PKR degradation [199], while PTV and SFSV NSs do not affect cellular levels of PKR [123,199].

Instead of degrading PKR, ANDV NP has developed another strategy. Indeed, in an overexpression system, ANDV NP inhibited PKR dimerization which is necessary for its activation [200], as seen for HCV NS5A protein [201]. HCV NS5A was found to bind to the dimerization site of PKR; in contrast, ANDV NP did not bind PKR. Thus, it remains to be understood how NP inhibits PKR dimerization.

#### 7.4.2. Inhibition of Tetherin

As mentioned above, JUNV egress is restricted by tetherin, as shown by the retention of JUNV VLPs based on the expression of the Z protein by tetherin [111]. Interestingly, JUNV NP seems to antagonize tetherin’s antiviral action, as overexpression of NP was able to rescue JUNV VLPs production. Moreover, JUNV NP tetherin antagonism was shown to be broad-spectrum. Indeed, its overexpression also led to the production of EBOV VLPs based on the expression of the matrix protein VP40, which are sensible to tetherin restriction activity. However, exactly how JUNV NP antagonizes tetherin remains to be determined.

## 8. Concluding Remarks

A plethora of host factors have been described to inhibit bunyaviruses infections, through mechanisms that vary depending on the host proteins but also on the targeted virus. Many restriction factors display a broad-spectrum antiviral activity, such as IFITMs, PKR, viperin or tetherin, as they target conserved features of virus replication such as virus entry, genomic replication, protein translation and virus egress. However, the role of hundreds of ISGs and host factors that are activated upon bunyavirus infection is still unknown. In the future, further genome-wide approaches, such as RNAi screens or CRISPR/Cas9 activation or knock-out screens will help to define important restriction factors that target bunyaviruses.

Gaining more knowledge about the strategies developed by bunyaviruses to escape the host antiviral state might be helpful to determine important and promising viral targets. Indeed, their inhibition could lead to the establishment of an efficient antiviral state that counteracts infection. The development of reverse genetic systems enabling the production of recombinant viruses or VLPs allowed the discovery of the role of some viral proteins, such as NSs of RVFV or the OTU domain of nairoviruses, which have a role in counteracting the innate immune responses. For phenuiviruses such as RVFV, whose NSs role in IFN-antagonism has been widely described, live attenuated virus vaccines in which NSs is deleted were developed and are much less virulent than the wild-type virus. The naturally attenuated mutant Clone 13 of RVFV has a deletion of 70% of its NSs gene and as many studies showed its immunogenicity and innocuity, it is therefore used as a live attenuated vaccine in livestock [202]. Other live attenuated vaccines are widely and safely used, such as the 17D strain of the yellow fever virus. One major obstacle of using live attenuated vaccines is the risk of their reversion to virulence, as seen for circulating vaccine-derived polioviruses (cVDPVs) [203]. However, Clone 13 does not revert to virulence upon experimental animal passage, suggesting that this risk under natural conditions is very low [204]. For CCHFV, it was recently shown that the stable occupancy of the CCHFV-encoded DUB with a Ub variant, blocking OTU DUB activity, blocked viral infection in addition to enhancing host antiviral responses [205]. Therefore, small antiviral molecules targeting its OTU domain could represent a promising area of antiviral research. These two examples provide elegant proofs of the great potential of targeting viral IFN-antagonists to design specific therapeutics or new efficient vaccines, and this area of research should be further developed.

## Dedication

The authors dedicate this paper to the memory of our colleague and friend Professor Renaud Mahieux (1968–2020).

## Figures and Tables

**Table 1:** Taxonomical classification of bunyaviruses discussed in this review.

Family	Genus	Species	Virus (Abbreviation)
*Arenaviridae*	*Mammarenavirus*	*Argentinian mammarenavirus*	Junín virus (JUNV)
		*Brazilian mammarenavirus*	Sabiá virus (SBAV)
		*Guanarito mammarenavirus*	Guanarito virus (GTOV)
		*Lassa mammarenavirus*	Lassa virus (LASV)
		*Lymphocytic choriomeningitis mammarenavirus*	Lymphocytic choriomeningitis virus (LCMV)
		*Machupo mammarenavirus*	Machupo virus (MACV)
*Hantaviridae*	*Orthohantavirus*	*Andes orthohantavirus*	Andes virus (ANDV)
		*Hantaan orthohantavirus*	Hantaan virus (HTNV)
		*Prospect Hill orthohantavirus*	Prospect Hill virus (PHV)
		*Puumala orthohantavirus*	Puumala orthohantavirus (PUUV)
		*Seoul orthohantavirus*	Seoul virus (SEOV)
		*Sin Nombre orthohantavirus*	New York virus (NYV)
			Sin Nombre virus (SNV)
		*Tula orthohantavirus*	Tula virus (TULV)
*Nairoviridae*	*Orthonairovirus*	*Crimean-Congo hemorrhagic fever orthonairovirus*	Crimean-Congo hemorrhagic fever virus (CCHFV)
		*Dugbe orthonairovirus*	Dugbe virus (DUGV)
		*Nairobi sheep disease* *orthonairovirus*	Nairobi sheep disease virus (NSDV)
		*Thiafora orthonairovirus*	Erve virus (ERVEV)
*Peribunyaviridae*	*Orthobunyavirus*	*Akabane orthobunyavirus*	Akabane virus (AKAV)
		*Bunyamwera orthobunyavirus*	Bunyamwera virus (BUNV)
		*Cache Valley orthobunyavirus*	Cache Valley virus (CVV)
		*La Crosse orthobunyavirus*	La Crosse virus (LACV)
		*Oropouche orthobynyavirus*	Oropouche virus (OROV)
		*Schmallenberg* *orthobunyavirus*	Sathuperi virus (SATV)
			Schmallenberg virus (SBV)
*Phenuiviridae*	*Banyangvirus*	*Guertu banyangvirus*	Guertu virus (GTV)
		*Heartland banyangvirus*	Heartland virus (HRTV)
		*Huaiyangshan banyangvirus*	Severe fever with thrombocytopenia syndrome virus (SFTSV)
	*Phlebovirus*	*Punta Toro phlebovirus*	Punta Toro virus (PTV)
		*Rift Valley fever phlebovirus*	Rift Valley fever virus (RVFV)
		*Sandfly fever Naples* *phlebovirus*	Sandfly fever Naples virus (SFNV)
			Toscana virus (TOSV)
		*Uukuniemi phlebovirus*	Uukuniemi virus (UUKV)

## References

[1] Abudurexiti A., Adkins S., Alioto D., Alkhovsky S.V., Avšič-Županc T., Ballinger M.J., Bente D.A., Beer M., Bergeron É., Blair C.D. (2019). Taxonomy of the Order Bunyavirales: Update 2019. Arch. Virol..

[2] Linero F.N., Sepúlveda C.S., Giovannoni F., Castilla V., García C.C., Scolaro L.A., Damonte E.B. (2012). Host Cell Factors as Antiviral Targets in Arenavirus Infection. Viruses.

[3] Elliott R.M., Weber F. (2009). Bunyaviruses and the Type I Interferon System. Viruses.

[4] Lasecka L., Baron M.D. (2014). The Molecular Biology of Nairoviruses, an Emerging Group of Tick-borne Arboviruses. Arch. Virol..

[5] Eifan S., Schnettler E., Dietrich I., Kohl A., Blomström A.-L. (2013). Non-Structural Proteins of Arthropod-Borne Bunyaviruses: Roles and Functions. Viruses.

[6] Wuerth J.D., Weber F. (2016). Phleboviruses and the Type I Interferon Response. Viruses.

[7] Prioritizing Diseases for Research and Development in Emergency Contexts. https://www.who.int/activities/prioritizing-diseases-for-research-and-development-in-emergency-contexts.

[8] Hughes H.R., Adkins S., Alkhovskiy S., Beer M., Blair C., Calisher C.H., Drebot M., Lambert A.J., De Souza W.M., Marklewitz M. (2020). ICTV Virus Taxonomy Profile: Peribunyaviridae. J. Gen. Virol..

[9] Garrison A.R., Alkhovsky С., Avšič-Županc T., Bente D.A., Bergeron É., Burt F., Di Paola N., Ergünay K., Hewson R., Kuhn J.H. (2020). ICTV Virus Taxonomy Profile: Nairoviridae. J. Gen. Virol..

[10] Walter C.T., Barr J.N. (2011). Recent Advances in the Molecular and Cellular Biology of Bunyaviruses. J. Gen. Virol..

[11] Radoshitzky S.R., Buchmeier M.J., Charrel R.N., Clegg J.C.S., Gonzalez J.-P.J., Günther S., Hepojoki J., Kuhn J.H., Lukashevich I.S., Romanowski V. (2019). ICTV Virus Taxonomy Profile: Arenaviridae. J. Gen. Virol..

[12] Schreur P.J.W., Kormelink R., Kortekaas J. (2018). Genome Packaging of the Bunyavirales. Curr. Opin. Virol..

[13] Muyangwa M., Martynova E.V., Khaiboullina S.F., Morzunov S.P., Rizvanov A.A. (2015). Hantaviral Proteins: Structure, Functions, and Role in Hantavirus Infection. Front. Microbiol..

[14] Albariño C.G., Bird B.H., Nichol S.T. (2007). A Shared Transcription Termination Signal on Negative and Ambisense RNA Genome Segments of Rift Valley Fever, Sandfly Fever Sicilian, and Toscana Viruses. J. Virol..

[15] Barnwal B., Karlberg H., Mirazimi A., Tan Y.-J. (2016). The Non-structural Protein of Crimean-Congo Hemorrhagic Fever Virus Disrupts the Mitochondrial Membrane Potential and Induces Apoptosis. J. Biol. Chem..

[16] Zivcec M., Scholte F.E.M., Spiropoulou C.F., Spengler J.R., Bergeron É. (2016). Molecular Insights into Crimean-Congo Hemorrhagic Fever Virus. Viruses.

[17] Pinschewer D.D., Pérez M., De La Torre J.C. (2005). Dual Role of the Lymphocytic Choriomeningitis Virus Intergenic Region in Transcription Termination and Virus Propagation. J. Virol..

[18] Olschewski S., Cusack S., Rosenthal M. (2020). The Cap-Snatching Mechanism of Bunyaviruses. Trends Microbiol..

[19] Kawasaki T., Kawai T. (2014). Toll-Like Receptor Signaling Pathways. Front. Immunol..

[20] Rehwinkel J., Gack M.U. (2020). RIG-I-like Receptors: Their Regulation and Roles in RNA Sensing. Nat. Rev. Immunol..

[21] Saito T., Gale M. (2008). Differential Recognition of Double-stranded RNA by RIG-I–like Receptors in Antiviral Immunity. J. Exp. Med..

[22] Huang C., Kolokoltsova O.A., Yun N.E., Seregin A.V., Poussard A.L., Walker A.G., Brasier A.R., Zhao Y., Tian B., De La Torre J.C. (2012). Junín Virus Infection Activates the Type I Interferon Pathway in a RIG-I-Dependent Manner. PLoS Negl. Trop. Dis..

[23] Min Y.-Q., Ning Y.-J., Wang H., Deng F. (2020). A RIG-I–like Receptor Directs Antiviral Responses to a Bunyavirus and is Antagonized by Virus-induced Blockade of TRIM25-mediated Ubiquitination. J. Biol. Chem..

[24] Habjan M., Andersson I., Klingström J., Schümann M., Martin A., Zimmermann P., Wagner V., Pichlmair A., Schneider U., Mühlberger E. (2008). Processing of Genome 5′ Termini as a Strategy of Negative-Strand RNA Viruses to Avoid RIG-I-Dependent Interferon Induction. PLoS ONE.

[25] Weber M., Gawanbacht A., Habjan M., Rang A., Borner C., Schmidt A.M., Veitinger S., Jacob R., Devignot S., Kochs G. (2013). Incoming RNA Virus Nucleocapsids Containing a 5′-Triphosphorylated Genome Activate RIG-I and Antiviral Signaling. Cell Host Microbe.

[26] Lee M.-H., Lalwani P., Raftery M.J., Matthaei M., Lütteke N., Kirsanovs S., Binder M., Ulrich R.G., Giese T., Wolff T. (2011). RNA Helicase Retinoic Acid-inducible Gene I as a Sensor of Hantaan Virus Replication. J. Gen. Virol..

[27] Spengler J.R., Patel J.R., Chakrabarti A.K., Zivcec M., García-Sastre A., Spiropoulou C.F., Bergeron É. (2015). RIG-I Mediates an Antiviral Response to Crimean-Congo Hemorrhagic Fever Virus. J. Virol..

[28] Zhou S., Cerny A.M., Zacharia A., Fitzgerald K.A., Kurt-Jones E.A., Finberg R.W. (2010). Induction and Inhibition of Type I Interferon Responses by Distinct Components of Lymphocytic Choriomeningitis Virus. J. Virol..

[29] Handke W., Oelschlegel R., Franke R., Krüger D.H., Rang A. (2009). Hantaan Virus Triggers TLR3-Dependent Innate Immune Responses. J. Immunol..

[30] Macal M., Lewis G.M., Kunz S., Flavell R., Harker J.A., Zúñiga E.I. (2012). Plasmacytoid Dendritic Cells Are Productively Infected and Activated through TLR-7 Early after Arenavirus Infection. Cell Host Microbe.

[31] Cuevas C.D., Lavanya M., Wang E., Ross S.R. (2011). Junin Virus Infects Mouse Cells and Induces Innate Immune Responses. J. Virol..

[32] Schneider W.M., Chevillotte M.D., Rice C.M. (2014). Interferon-Stimulated Genes: A Complex Web of Host Defenses. Annu. Rev. Immunol..

[33] Lazear H.M., Schoggins J.W., Diamond M.S. (2019). Shared and Distinct Functions of Type I and Type III Interferons. Immunity.

[34] Garrison A.R., Smith D.R., Golden J.W. (2019). Animal Models for Crimean-Congo Hemorrhagic Fever Human Disease. Viruses.

[35] Bouloy M., Janzen C., Vialat P., Khun H., Pavlovic J., Huerre M., Haller O. (2001). Genetic Evidence for an Interferon-Antagonistic Function of Rift Valley Fever Virus Nonstructural Protein NSs. J. Virol..

[36] Mendenhall M., Wong M.-H., Skirpstunas R., Morrey J.D., Gowen B.B. (2009). Punta Toro Virus (Bunyaviridae, Phlebovirus) Infection in Mice: Strain Differences in Pathogenesis and Host Interferon Response. Virology.

[37] Blakqori G., Delhaye S., Habjan M., Blair C.D., Sánchez-Vargas I., Olson K.E., Attarzadeh-Yazdi G., Fragkoudis R., Kohl A., Kalinke U. (2007). La Crosse Bunyavirus Nonstructural Protein NSs Serves to Suppress the Type I Interferon System of Mammalian Hosts. J. Virol..

[38] Boyd A., Fazakerley J.K., Bridgen A. (2006). Pathogenesis of Dugbe Virus Infection in Wild-type and Interferon-deficient Mice. J. Gen. Virol..

[39] Hefti H.P., Frese M., Landis H., Di Paolo C., Aguzzi A., Haller O., Pavlovic J. (1999). Human MxA Protein Protects Mice Lacking a Functional Alpha/Beta Interferon System against La Crosse Virus and Other Lethal Viral Infections. J. Virol..

[40] Wichmann D., Grone H.-J., Frese M., Pavlovic J., Anheier B., Haller O., Klenk H.-D., Feldmann H. (2002). Hantaan Virus Infection Causes an Acute Neurological Disease That Is Fatal in Adult Laboratory Mice. J. Virol..

[41] Kell A.M., Hemann E.A., Turnbull J.B., Gale M. (2020). RIG-I-like Receptor Activation Drives Type I IFN and Antiviral Signaling to Limit Hantaan Orthohantavirus Replication. PLoS Pathog..

[42] Hawman D.W., Meade-White K., Leventhal S., Feldmann F., Okumura A., Smith B., Scott D., Feldmann H. (2021). Immunocompetent Mouse Model for Crimean-Congo Hemorrhagic Fever Virus. eLife.

[43] Pinkham C., Dahal B., De La Fuente C.L., Bracci N., Beitzel B., Lindquist M., Garrison A., Schmaljohn C., Palacios G., Narayanan A. (2017). Alterations in the Host Transcriptome in vitro Following Rift Valley Fever Virus Infection. Sci. Rep..

[44] Havranek K.E., White L.A., Lanchy J.-M., Lodmell J.S. (2019). Transcriptome Profiling in Rift Valley Fever Virus Infected Cells Reveals Modified Transcriptional and Alternative Splicing Programs. PLoS ONE.

[45] Blomström A.-L., Gu Q., Barry G., Wilkie G., Skelton J.K., Baird M., McFarlane M., Schnettler E., Elliott R.M., Palmarini M. (2015). Transcriptome Analysis Reveals the Host Response to Schmallenberg Virus in Bovine Cells and Antagonistic Effects of the NSs Protein. BMC Genom..

[46] Malhotra S., Yen J.Y., Honko A.N., Garamszegi S., Caballero I.S., Johnson J.C., Mucker E.M., Trefry J.C., Hensley L.E., Connor J.H. (2013). Transcriptional Profiling of the Circulating Immune Response to Lassa Virus in an Aerosol Model of Exposure. PLoS Negl. Trop. Dis..

[47] Zapata J.C., Carrion R., Patterson J.L., Crasta O., Zhang Y., Mani S., Jett M., Poonia B., Djavani M., White D.M. (2013). Transcriptome Analysis of Human Peripheral Blood Mononuclear Cells Exposed to Lassa Virus and to the Attenuated Mopeia/Lassa Reassortant 29 (ML29), a Vaccine Candidate. PLoS Negl. Trop. Dis..

[48] Diamond M.S., Farzan M. (2012). The Broad-spectrum Antiviral Functions of IFIT and IFITM Proteins. Nat. Rev. Immunol..

[49] Zhao X., Li J., Winkler C.A., An P., Guo J.-T. (2019). IFITM Genes, Variants, and Their Roles in the Control and Pathogenesis of Viral Infections. Front. Microbiol..

[50] Mudhasani R., Tran J.P., Retterer C., Radoshitzky S.R., Kota K.P., Altamura L.A., Smith J.M., Packard B.Z., Kuhn J.H., Costantino J. (2013). IFITM-2 and IFITM-3 but Not IFITM-1 Restrict Rift Valley Fever Virus. J. Virol..

[51] Xu-Yang Z., Pei-Yu B., Chuan-Tao Y., Wei Y., Hong-Wei M., Kang T., Chun-Mei Z., Ying-Feng L., Xin W., Ping-Zhong W. (2017). Interferon-Induced Transmembrane Protein 3 Inhibits Hantaan Virus Infection, and Its Single Nucleotide Polymorphism rs12252 Influences the Severity of Hemorrhagic Fever with Renal Syndrome. Front. Immunol..

[52] Jegado B., Journo C., Mahieux R. (2018). Un Double Effet Antiviral des IFITM sur les Virus Enveloppés. Médecine Sci..

[53] Feng J., Wickenhagen A., Turnbull M.L., Rezelj V.V., Kreher F., Tilston-Lunel N.L., Slack G.S., Brennan B., Koudriakova E., Shaw A.E. (2018). Interferon-Stimulated Gene (ISG)-Expression Screening Reveals the Specific Antibunyaviral Activity of ISG20. J. Virol..

[54] Brass A.L., Huang I.-C., Benita Y., John S.P., Krishnan M.N., Feeley E.M., Ryan B.J., Weyer J.L., Van Der Weyden L., Fikrig E. (2009). The IFITM Proteins Mediate Cellular Resistance to Influenza A H1N1 Virus, West Nile Virus, and Dengue Virus. Cell.

[55] Wang S., Chi X., Wei H., Chen Y., Chen Z., Huang S., Chen J.-L. (2014). Influenza A Virus-Induced Degradation of Eukaryotic Translation Initiation Factor 4B Contributes to Viral Replication by Suppressing IFITM3 Protein Expression. J. Virol..

[56] Wang B., Lam T.H., Soh M.K., Ye Z., Chen J., Ren E.C. (2018). Influenza A Virus Facilitates Its Infectivity by Activating p53 to Inhibit the Expression of Interferon-Induced Transmembrane Proteins. Front. Immunol..

[57] Chen D., Hou Z., Jiang D., Zheng M., Li G., Zhang Y., Li R., Lin H., Chang J., Zeng H. (2019). GILT Restricts the Cellular Entry Mediated by the Envelope Glycoproteins of SARS-CoV, Ebola Virus and Lassa Fever Virus. Emerg. Microbes Infect..

[58] Zhao J., Chen J., Li M., Chen M., Sun C. (2020). Multifaceted Functions of CH25H and 25HC to Modulate the Lipid Metabolism, Immune Responses, and Broadly Antiviral Activities. Viruses.

[59] Liu S.-Y., Aliyari R., Chikere K., Li G., Marsden M.D., Smith J.K., Pernet O., Guo H., Nusbaum R., Zack J.A. (2013). Interferon-Inducible Cholesterol-25-Hydroxylase Broadly Inhibits Viral Entry by Production of 25-Hydroxycholesterol. Immunity.

[60] Shrivastava-Ranjan P., Bergeron E., Chakrabarti A.K., Albariño C.G., Flint M., Nichol S.T., Spiropoulou C.F. (2016). 25-Hydroxycholesterol Inhibition of Lassa Virus Infection through Aberrant GP1 Glycosylation. MBio.

[61] Simon M., Johansson C., Mirazimi A. (2009). Crimean-Congo Hemorrhagic Fever Virus Entry and Replication is Clathrin-, pH- and Cholesterol-dependent. J. Gen. Virol..

[62] Sadler A.J., Williams B.R.G. (2008). Interferon-inducible Antiviral Effectors. Nat. Rev. Immunol..

[63] Frese M., Kochs G., Feldmann H., Hertkorn C., Haller O. (1996). Inhibition of Bunyaviruses, Phleboviruses, and Hantaviruses by Human MxA Protein. J. Virol..

[64] Reichelt M., Stertz S., Krijnse-Locker J., Haller O., Kochs G. (2004). Missorting of LaCrosse Virus Nucleocapsid Protein by the Interferon-induced MxA GTPase Involves Smooth ER Membranes. Traffic.

[65] Kochs G., Janzen C., Hohenberg H., Haller O. (2002). Antivirally Active MxA Protein Sequesters La Crosse Virus Nucleocapsid Protein into Perinuclear Complexes. Proc. Natl. Acad. Sci. USA.

[66] Miura T.A., Carlson J.O., Beaty B.J., Bowen R.A., Olson K.E. (2001). Expression of Human MxA Protein in Mosquito Cells Interferes with LaCrosse Virus Replication. J. Virol..

[67] Sandrock M., Frese M., Haller O., Kochs G. (2001). Interferon-Induced Rat Mx Proteins Confer Resistance to Rift Valley Fever Virus and Other Arthropod-Borne Viruses. J. Interf. Cytokine Res..

[68] Andersson I., Bladh L., Mousavi-Jazi M., Magnusson K.-E., Lundkvist A., Haller O., Mirazimi A. (2004). Human MxA Protein Inhibits the Replication of Crimean-Congo Hemorrhagic Fever Virus. J. Virol..

[69] Bridgen A., Dalrymple D.A., Weber F., Elliott R.M. (2004). Inhibition of Dugbe Nairovirus Replication by Human MxA Protein. Virus Res..

[70] Nam J.-H., Hwang K.-A., Yu C.-H., Kang T.-H., Shin J.-Y., Choi W.-Y., Kim I.-B., Joo Y.-R., Cho H.-W., Park K.-Y. (2003). Expression of Interferon Inducible Genes Following Hantaan Virus Infection as a Mechanism of Resistance in A549 Cells. Virus Genes.

[71] Khaiboullina S.F., Rizvanov A.A., Otteson E., Miyazato A., Maciejewski J., Jeor S.S. (2004). Regulation of Cellular Gene Expression in Endothelial Cells by Sin Nombre and Prospect Hill Viruses. Viral Immunol..

[72] Khaiboullina S.F., Rizvanov A.A., Deyde V.M., Jeor S.C.S. (2004). Andes Virus Stimulates Interferon-inducible MxA Protein Expression in Endothelial Cells. J. Med. Virol..

[73] Kanerva M., Melén K., Vaheri A., Julkunen I. (1996). Inhibition of Puumala and Tula Hantaviruses in Vero Cells by MxA Protein. Virology.

[74] Oelschlegel R., Krüger D.H., Rang A. (2007). MxA-independent Inhibition of Hantaan Virus Replication Induced by Type I and Type II Interferon in vitro. Virus Res..

[75] Graf L., Dick A., Sendker F., Barth E., Marz M., Daumke O., Kochs G. (2018). Effects of Allelic Variations in the Human Myxovirus Resistance Protein A on Its Antiviral Activity. J. Biol. Chem..

[76] Habjan M., Penski N., Wagner V., Spiegel M., Överby A.K., Kochs G., Huiskonen J.T., Weber F. (2009). Efficient Production of Rift Valley Fever Virus-like Particles: The Antiviral Protein MxA Can Inhibit Primary Transcription of Bunyaviruses. Virology.

[77] Carlton-Smith C., Elliott R.M. (2012). Viperin, MTAP44, and Protein Kinase R Contribute to the Interferon-Induced Inhibition of Bunyamwera Orthobunyavirus Replication. J. Virol..

[78] Streitenfeld H., Boyd A., Fazakerley J.K., Bridgen A., Elliott R.M., Weber F. (2003). Activation of PKR by Bunyamwera Virus Is Independent of the Viral Interferon Antagonist NSs. J. Virol..

[79] Habjan M., Pichlmair A., Elliott R.M., Overby A.K., Glatter T., Gstaiger M., Superti-Furga G., Unger H., Weber F. (2009). NSs Protein of Rift Valley Fever Virus Induces the Specific Degradation of the Double-Stranded RNA-Dependent Protein Kinase. J. Virol..

[80] Ikegami T., Narayanan K., Won S., Kamitani W., Peters C.J., Makino S. (2009). Rift Valley Fever Virus NSs Protein Promotes Post-Transcriptional Downregulation of Protein Kinase PKR and Inhibits eIF2α Phosphorylation. PLoS Pathog..

[81] Huang C., Kolokoltsova O.A., Mateer E.J., Koma T., Paessler S. (2017). Highly Pathogenic New World Arenavirus Infection Activates the Pattern Recognition Receptor Protein Kinase R without Attenuating Virus Replication in Human Cells. J. Virol..

[82] Pichlmair A., Lassnig C., Eberle C.-A., Górna M.W., Baumann C.L., Burkard T.R., Bürckstümmer T., Stefanovic A., Krieger S., Bennett K.L. (2011). IFIT1 is an Antiviral Protein That Recognizes 5′-triphosphate RNA. Nat. Immunol..

[83] Atasheva S., Akhrymuk M., Frolova E.I., Frolov I. (2012). New PARP Gene with an Anti-Alphavirus Function. J. Virol..

[84] Atasheva S., Frolova E.I., Frolov I. (2014). Interferon-Stimulated Poly(ADP-Ribose) Polymerases Are Potent Inhibitors of Cellular Translation and Virus Replication. J. Virol..

[85] Hopkins K.C., McLane L.M., Maqbool T., Panda D., Gordesky-Gold B., Cherry S. (2013). A Genome-wide RNAi Screen Reveals That mRNA Decapping Restricts Bunyaviral Replication by Limiting the Pools of Dcp2-accessible Targets for Cap-snatching. Genes Dev..

[86] Ma X., Zhou Y., Moffett P. (2019). Alterations in Cellular RNA Decapping Dynamics Affect Tomato Spotted Wilt Virus Cap Snatching and Infection in *Arabidopsis*. New Phytol..

[87] Jin H., Elliott R.M. (1993). Non-viral Sequences at the 5’ Ends of Dugbe Nairovirus S mRNAs. J. Gen. Virol..

[88] Decroly E., Ferron F., Lescar J., Canard B. (2011). Conventional and Unconventional Mechanisms for Capping Viral mRNA. Nat. Rev. Genet..

[89] Moy R.H., Cole B.S., Yasunaga A., Gold B., Shankarling G., Varble A., Molleston J.M., Tenoever B.R., Lynch K.W., Cherry S. (2014). Stem-Loop Recognition by DDX17 Facilitates miRNA Processing and Antiviral Defense. Cell.

[90] Nelson C.R., Mrozowich T., Park S.M., D’Souza S., Henrickson A., Vigar J.R.J., Wieden H.-J., Owens R.J., Demeler B., Patel T.R. (2020). Human DDX17 Unwinds Rift Valley Fever Virus Non-Coding RNAs. Int. J. Mol. Sci..

[91] DeDiego M.L., Nogales A., Martinez-Sobrido L., Topham D.J. (2019). Interferon-Induced Protein 44 Interacts with Cellular FK506-Binding Protein 5, Negatively Regulates Host Antiviral Responses, and Supports Virus Replication. MBio.

[92] Djavani M., Rodas J., Lukashevich I.S., Horejsh D., Pandolfi P.P., Borden K.L.B., Salvato M.S. (2001). Role of the Promyelocytic Leukemia Protein PML in the Interferon Sensitivity of Lymphocytic Choriomeningitis Virus. J. Virol..

[93] Bonilla W.V., Pinschewer D.D., Klenerman P., Rousson V., Gaboli M., Pandolfi P.P., Zinkernagel R.M., Salvato M.S., Hengartner H. (2002). Effects of Promyelocytic Leukemia Protein on Virus-Host Balance. J. Virol..

[94] Borden K.L.B., Dwyer E.J.C., Salvato M.S. (1998). An Arenavirus RING (Zinc-Binding) Protein Binds the Oncoprotein Promyelocyte Leukemia Protein (PML) and Relocates PML Nuclear Bodies to the Cytoplasm. J. Virol..

[95] Wang X., Han Y., Dang Y., Fu W., Zhou T., Ptak R.G., Zheng Y.-H. (2010). Moloney Leukemia Virus 10 (MOV10) Protein Inhibits Retrovirus Replication. J. Biol. Chem..

[96] Liu T., Sun Q., Liu Y., Cen S., Zhang Q. (2019). The MOV10 Helicase Restricts Hepatitis B Virus Replication by Inhibiting Viral Reverse Transcription. J. Biol. Chem..

[97] Schoggins J.W., Wilson S.J., Panis M., Murphy M.Y., Jones C.T., Bieniasz P.D., Rice C.M. (2011). A Diverse Range of Gene Products Are Effectors of the Type I Interferon Antiviral Response. Nat. Cell Biol..

[98] Cuevas R.A., Ghosh A., Wallerath C., Hornung V., Coyne C.B., Sarkar S.N. (2016). MOV10 Provides Antiviral Activity against RNA Viruses by Enhancing RIG-I-MAVS-Independent IFN Induction. J. Immunol..

[99] Zhang J., Huang F., Tan L., Bai C., Chen B., Liu J., Liang J., Liu C., Zhang S., Lu G. (2016). Host Protein Moloney Leukemia Virus 10 (MOV10) Acts as a Restriction Factor of Influenza A Virus by Inhibiting the Nuclear Import of the Viral Nucleoprotein. J. Virol..

[100] Haussecker D., Cao D., Huang Y., Parameswaran P., Fire A.Z., Kay M.A. (2008). Capped Small RNAs and MOV10 in Human Hepatitis Delta Virus Replication. Nat. Struct. Mol. Biol..

[101] Mo Q., Xu Z., Deng F., Wang H., Ning Y.-J. (2020). Host Restriction of Emerging High-pathogenic Bunyaviruses via MOV10 by Targeting Viral Nucleoprotein and Blocking Ribonucleoprotein Assembly. PLoS Pathog..

[102] Zhou Z., Wang N., Woodson S.E., Dong Q., Wang J., Liang Y., Rijnbrand R., Wei L., Nichols J.E., Guo J.-T. (2011). Antiviral Activities of ISG20 in Positive-strand RNA Virus Infections. Virology.

[103] Leong C.R., Funami K., Oshiumi H., Mengao D., Takaki H., Matsumoto M., Aly H.H., Watashi K., Chayama K., Seya T. (2016). Interferon-stimulated Gene of 20 kDa Protein (ISG20) Degrades RNA of Hepatitis B Virus to Impede the Replication of HBV in vitro and in vivo. Oncotarget.

[104] Valle T.Z.D., Billecocq A., Guillemot L., Alberts R., Gommet C., Geffers R., Calabrese K., Schughart K., Bouloy M., Montagutelli X. (2010). A New Mouse Model Reveals a Critical Role for Host Innate Immunity in Resistance to Rift Valley Fever. J. Immunol..

[105] Hinson E.R., Joshi N.S., Chen J.H., Rahner C., Jung Y.W., Wang X., Kaech S.M., Cresswell P. (2010). Viperin Is Highly Induced in Neutrophils and Macrophages during Acute and Chronic Lymphocytic Choriomeningitis Virus Infection. J. Immunol..

[106] Cárcamo J.R.P., Morell M.L., Vázquez C.A., Vatansever S., Upadhyay A.S., Överby A.K., Cordo S.M., García C.C. (2018). The Interplay between Viperin Antiviral Activity, Lipid Droplets and Junín Mammarenavirus Multiplication. Virology.

[107] Perez-Caballero D., Zang T., Ebrahimi A., McNatt M.W., Gregory D.A., Johnson M.C., Bieniasz P.D. (2009). Tetherin Inhibits HIV-1 Release by Directly Tethering Virions to Cells. Cell.

[108] Swiecki M., Omattage N.S., Brett T.J. (2013). BST-2/tetherin: Structural Biology, Viral Antagonism, and Immunobiology of a Potent Host Antiviral Factor. Mol. Immunol..

[109] Radoshitzky S.R., Dong L., Chi X., Clester J.C., Retterer C., Spurgers K., Kuhn J.H., Sandwick S., Ruthel G., Kota K. (2010). Infectious Lassa Virus, but Not Filoviruses, Is Restricted by BST-2/Tetherin. J. Virol..

[110] Sakuma T., Noda T., Urata S., Kawaoka Y., Yasuda J. (2008). Inhibition of Lassa and Marburg Virus Production by Tetherin. J. Virol..

[111] Zadeh V.R., Urata S., Sakaguchi M., Yasuda J. (2020). Human BST-2/Tetherin Inhibits Junin Virus Release from Host Cells and Its Inhibition is Partially Counteracted by Viral Nucleoprotein. J. Gen. Virol..

[112] Sakuma T., Sakurai A., Yasuda J. (2009). Dimerization of Tetherin Is Not Essential for Its Antiviral Activity against Lassa and Marburg Viruses. PLoS ONE.

[113] Varela M., Piras I.M., Mullan C., Shi X., Tilston-Lunel N.L., Pinto R.M., Taggart A., Welch S.R., Neil S.J., Kreher F. (2017). Sensitivity to BST-2 Restriction Correlates with Orthobunyavirus Host Range. Virology.

[114] Weber F., Wagner V., Rasmussen S.B., Hartmann R., Paludan S.R. (2006). Double-Stranded RNA Is Produced by Positive-Strand RNA Viruses and DNA Viruses but Not in Detectable Amounts by Negative-Strand RNA Viruses. J. Virol..

[115] Mir M.A., Panganiban A.T. (2005). The Hantavirus Nucleocapsid Protein Recognizes Specific Features of the Viral RNA Panhandle and Is Altered in Conformation upon RNA Binding. J. Virol..

[116] Mateer E.J., Maruyama J., Card G.E., Paessler S., Huang C. (2020). Lassa Virus, but Not Highly Pathogenic New World Arenaviruses, Restricts Immunostimulatory Double-Stranded RNA Accumulation during Infection. J. Virol..

[117] Hastie K.M., Kimberlin C.R., Zandonatti M.A., Macrae I.J., Saphire E.O. (2011). Structure of the Lassa Virus Nucleoprotein Reveals a dsRNA-specific 3’ to 5’ Exonuclease Activity Essential for Immune Suppression. Proc. Natl. Acad. Sci. USA.

[118] Reynard S., Russier M., Fizet A., Carnec X., Baize S. (2014). Exonuclease Domain of the Lassa Virus Nucleoprotein Is Critical to Avoid RIG-I Signaling and To Inhibit the Innate Immune Response. J. Virol..

[119] Jiang X., Huang Q., Wang W., Dong H., Ly H., Liang Y., Dong C. (2013). Structures of Arenaviral Nucleoproteins with Triphosphate dsRNA Reveal a Unique Mechanism of Immune Suppression. J. Biol. Chem..

[120] Vialat P., Billecocq A., Kohl A., Bouloy M. (2000). The S Segment of Rift Valley Fever Phlebovirus (Bunyaviridae) Carries Determinants for Attenuation and Virulence in Mice. J. Virol..

[121] Perrone L.A., Narayanan K., Worthy M., Peters C.J. (2006). The S Segment of Punta Toro Virus (Bunyaviridae, Phlebovirus) Is a Major Determinant of Lethality in the Syrian Hamster and Codes for a Type I Interferon Antagonist. J. Virol..

[122] Savellini G.G., Weber F., Terrosi C., Habjan M., Martorelli B., Cusi M.G. (2010). Toscana Virus Induces Interferon Although Its NSs Protein Reveals Antagonistic Activity. J. Gen. Virol..

[123] Lihoradova O.A., Indran S.V., Kalveram B., Lokugamage N., Head J.A., Gong B., Tigabu B., Juelich T.L., Freiberg A.N., Ikegami T. (2013). Characterization of Rift Valley Fever Virus MP-12 Strain Encoding NSs of Punta Toro Virus or Sandfly Fever Sicilian Virus. PLoS Negl. Trop. Dis..

[124] Zhang S., Zheng B., Wang T., Li A., Wan J., Qu J., Li C., Li D., Liang M. (2017). NSs Protein of Severe Fever with Thrombocytopenia Syndrome Virus Suppresses Interferon Production through Different Mechanism Than Rift Valley Fever Virus. Acta Virol..

[125] Kohl A., Clayton R.F., Weber F., Bridgen A., Randall R.E., Elliott R.M. (2003). Bunyamwera Virus Nonstructural Protein NSs Counteracts Interferon Regulatory Factor 3-Mediated Induction of Early Cell Death. J. Virol..

[126] Thomas D., Blakqori G., Wagner V., Banholzer M., Kessler N., Elliott R.M., Haller O., Weber F. (2004). Inhibition of RNA Polymerase II Phosphorylation by a Viral Interferon Antagonist. J. Biol. Chem..

[127] Tilston-Lunel N.L., Acrani G.O., Randall R.E., Elliott R.M. (2015). Generation of Recombinant Oropouche Viruses Lacking the Nonstructural Protein NSm or NSs. J. Virol..

[128] Barry G., Varela M., Ratinier M., Blomström A.-L., Caporale M., Seehusen F., Hahn K., Schnettler E., Baumgärtner W., Kohl A. (2014). NSs Protein of Schmallenberg Virus Counteracts the Antiviral Response of the Cell by Inhibiting Its Transcriptional Machinery. J. Gen. Virol..

[129] Jääskeläinen K.M., Kaukinen P., Minskaya E.S., Plyusnina A., Vapalahti O., Elliott R.M., Weber F., Vaheri A., Plyusnin A. (2007). Tula and Puumala Hantavirus NSs ORFs are Functional and the Products Inhibit Activation of the Interferon-beta Promoter. J. Med. Virol..

[130] Vera-Otarola J., Solis L., Lowy F., Olguín V., Angulo J., Pino K., Tischler N.D., Otth C., Padula P., López-Lastra M. (2020). The Andes Orthohantavirus NSs Protein Antagonizes the Type I Interferon Response by Inhibiting MAVS Signaling. J. Virol..

[131] Ikegami T., Narayanan K., Won S., Kamitani W., Peters C.J., Makino S. (2009). Dual Functions of Rift Valley Fever Virus NSs Protein: Inhibition of Host mRNA Transcription and Post-transcriptional Downregulation of Protein Kinase PKR. Ann. N. Y. Acad. Sci..

[132] Le May N., Dubaele S., De Santis L.P., Billecocq A., Bouloy M., Egly J.-M. (2004). TFIIH Transcription Factor, a Target for the Rift Valley Hemorrhagic Fever Virus. Cell.

[133] Cyr N., De La Fuente C., Lecoq L., Guendel I., Chabot P.R., Kehn-Hall K., Omichinski J.G. (2015). A ΩXaV motif in the Rift Valley fever virus NSs protein is essential for degrading p62, forming nuclear filaments and virulence. Proc. Natl. Acad. Sci. USA.

[134] Kalveram B., Lihoradova O., Ikegami T. (2011). NSs Protein of Rift Valley Fever Virus Promotes Posttranslational Downregulation of the TFIIH Subunit p62. J. Virol..

[135] Kainulainen M., Habjan M., Hubel P., Busch L., Lau S., Colinge J., Superti-Furga G., Pichlmair A., Weber F. (2014). Virulence Factor NSs of Rift Valley Fever Virus Recruits the F-Box Protein FBXO3 To Degrade Subunit p62 of General Transcription Factor TFIIH. J. Virol..

[136] Copeland A.M., Van Deusen N.M., Schmaljohn C.S. (2015). Rift Valley Fever Virus NSS Gene Expression Correlates with a Defect in Nuclear mRNA Export. Virology.

[137] Le May N., Mansuroglu Z., Léger P., Josse T., Blot G., Billecocq A., Flick R., Jacob Y., Bonnefoy E., Bouloy M. (2008). A SAP30 Complex Inhibits IFN-β Expression in Rift Valley Fever Virus Infected Cells. PLoS Pathog..

[138] Rezelj V.V., Överby A.K., Elliott R.M. (2015). Generation of Mutant Uukuniemi Viruses Lacking the Nonstructural Protein NSs by Reverse Genetics Indicates that NSs Is a Weak Interferon Antagonist. J. Virol..

[139] Simons J.F., Hellman U., Pettersson R.F. (1990). Uukuniemi Virus S RNA Segment: Ambisense Coding Strategy, Packaging of Complementary Strands into Virions, and Homology to Members of the Genus Phlebovirus. J. Virol..

[140] Ikegami T., Won S., Peters C.J., Makino S. (2005). Rift Valley Fever Virus NSs mRNA Is Transcribed from an Incoming Anti-Viral-Sense S RNA Segment. J. Virol..

[141] Brennan B., Li P., Zhang S., Li A., Liang M., Li D., Elliott R.M. (2014). Reverse Genetics System for Severe Fever with Thrombocytopenia Syndrome Virus. J. Virol..

[142] Verbruggen P., Ruf M., Blakqori G., Överby A.K., Heidemann M., Eick D., Weber F. (2011). Interferon Antagonist NSs of La Crosse Virus Triggers a DNA Damage Response-like Degradation of Transcribing RNA Polymerase II. J. Biol. Chem..

[143] Schoen A., Lau S., Verbruggen P., Weber F. (2020). Elongin C Contributes to RNA Polymerase II Degradation by the Interferon Antagonist NSs of La Crosse Orthobunyavirus. J. Virol..

[144] Gouzil J., Fablet A., Lara E., Caignard G., Cochet M., Kundlacz C., Palmarini M., Varela M., Breard E., Sailleau C. (2017). Nonstructural Protein NSs of Schmallenberg Virus Is Targeted to the Nucleolus and Induces Nucleolar Disorganization. J. Virol..

[145] Heaton S.M., Borg N.A., Dixit V.M. (2016). Ubiquitin in the Activation and Attenuation of Innate Antiviral Immunity. J. Exp. Med..

[146] Hu H., Sun S.-C. (2016). Ubiquitin Signaling in Immune Responses. Cell Res..

[147] Gack M.U., Shin Y.C., Joo C.-H., Urano T., Liang C., Sun L., Takeuchi O., Akira S., Chen Z., Inoue S. (2007). TRIM25 RING-finger E3 Ubiquitin Ligase is Essential for RIG-I-mediated Antiviral Activity. Nat. Cell Biol..

[148] Wertz I.E., O’Rourke K.M., Zhou H., Eby M., Aravind L., Seshagiri S., Wu P., Wiesmann C., Baker R.T., Boone D.L. (2004). De-ubiquitination and Ubiquitin Ligase Domains of A20 Downregulate NF-κB Signalling. Nat. Cell Biol..

[149] Kayagaki N., Phung Q., Chan S., Chaudhari R., Quan C., O’Rourke K.M., Eby M., Pietras E., Cheng G., Bazan J.F. (2007). DUBA: A Deubiquitinase That Regulates Type I Interferon Production. Science.

[150] Kinsella E., Martin S.G., Grolla A., Czub M., Feldmann H., Flick R. (2004). Sequence Determination of the Crimean–Congo Hemorrhagic Fever Virus L Segment. Virology.

[151] Honig J.E., Osborne J.C., Nichol S.T. (2004). Crimean–Congo Hemorrhagic Fever Virus Genome L RNA Segment and Encoded Protein. Virology.

[152] Walker P.J., Widen S.G., Wood T.G., Guzman H., Tesh R.B., Vasilakis N. (2016). A Global Genomic Characterization of Nairoviruses Identifies Nine Discrete Genogroups with Distinctive Structural Characteristics and Host-Vector Associations. Am. J. Trop. Med. Hyg..

[153] Zhang H.-M., Yang J., Sun H.-R., Xin X., Wang H.-D., Chen J.-P., Adams M.J. (2007). Genomic Analysis of Rice Stripe Virus Zhejiang Isolate Shows the Presence of an OTU-like Domain in the RNA1 Protein and a Novel Sequence Motif Conserved within the Intergenic Regions of Ambisense Segments of Tenuiviruses. Arch. Virol..

[154] Zhao S., Gu X., Li J., Liang C. (2021). The N-terminal Cysteine Protease Domain of Rice Stripe Tenuivirus Pc1 Possesses Deubiquitinating Enzyme Activity. Virus Genes.

[155] Frias-Staheli N., Giannakopoulos N.V., Kikkert M., Taylor S.L., Bridgen A., Paragas J., Richt J.A., Rowland R.R., Schmaljohn C.S., Lenschow D.J. (2007). Ovarian Tumor Domain-Containing Viral Proteases Evade Ubiquitin- and ISG15-Dependent Innate Immune Responses. Cell Host Microbe.

[156] Holzer B., Bakshi S., Bridgen A., Baron M.D. (2011). Inhibition of Interferon Induction and Action by the Nairovirus Nairobi Sheep Disease Virus/Ganjam Virus. PLoS ONE.

[157] Bakshi S., Holzer B., Bridgen A., McMullan G., Quinn D.G., Baron M.D. (2013). Dugbe Virus Ovarian Tumour Domain Interferes with Ubiquitin/ISG15-regulated Innate Immune Cell Signalling. J. Gen. Virol..

[158] Kocabas F., Aslan G.S. (2015). Fluorometric CCHFV OTU Protease Assay with Potent Inhibitors. Virus Genes.

[159] Scholte F.E., Zivcec M., Dzimianski J.V., Deaton M.K., Spengler J.R., Welch S.R., Nichol S.T., Pegan S.D., Spiropoulou C.F., Bergeron É. (2017). Crimean-Congo Hemorrhagic Fever Virus Suppresses Innate Immune Responses via a Ubiquitin and ISG15 Specific Protease. Cell Rep..

[160] Akutsu M., Ye Y., Virdee S., Chin J.W., Komander D. (2011). Molecular Basis for Ubiquitin and ISG15 Cross-reactivity in Viral Ovarian Tumor Domains. Proc. Natl. Acad. Sci. USA.

[161] Capodagli G.C., McKercher M.A., Baker E.A., Masters E.M., Brunzelle J.S., Pegan S.D. (2011). Structural Analysis of a Viral Ovarian Tumor Domain Protease from the Crimean-Congo Hemorrhagic Fever Virus in Complex with Covalently Bonded Ubiquitin. J. Virol..

[162] Van Kasteren P., Beugeling C., Ninaber D., Frias-Staheli N., Van Boheemen S., García-Sastre A., Snijder E., Kikkert M. (2011). Arterivirus and Nairovirus Ovarian Tumor Domain-Containing Deubiquitinases Target Activated RIG-I To Control Innate Immune Signaling. J. Virol..

[163] Capodagli G.C., Deaton M.K., Baker E.A., Lumpkin R.J., Pegan S.D. (2013). Diversity of Ubiquitin and ISG15 Specificity among Nairoviruses’ Viral Ovarian Tumor Domain Proteases. J. Virol..

[164] Deaton M.K., Dzimianski J.V., Daczkowski C.M., Whitney G.K., Mank N.J., Parham M.M., Bergeron E., Pegan S.D. (2016). Biochemical and Structural Insights into the Preference of Nairoviral DeISGylases for Interferon-Stimulated Gene Product 15 Originating from Certain Species. J. Virol..

[165] Dzimianski J.V., Beldon B.S., Daczkowski C.M., Goodwin O.Y., Scholte F.E.M., Bergeron É., Pegan S.D. (2019). Probing the Impact of Nairovirus Genomic Diversity on Viral Ovarian Tumor Domain Protease (vOTU) Structure and Deubiquitinase Activity. PLoS Pathog..

[166] Devignot S., Kromer T., Mirazimi A., Weber F. (2020). ISG15 Overexpression Compensates the Defect of Crimean-Congo Hemorrhagic Fever Virus Polymerase Bearing a Protease-inactive Ovarian Tumor Domain. PLoS Neglected Trop. Dis..

[167] Haas A.L., Ahrens P., Bright P.M., Ankel H. (1987). Interferon Induces a 15-kilodalton Protein Exhibiting Marked Homology to Ubiquitin. J. Biol. Chem..

[168] Loeb K.R., Haas A.L. (1992). The Interferon-inducible 15-kDa Ubiquitin Homolog Conjugates to Intracellular Proteins. J. Biol. Chem..

[169] Perng Y.-C., Lenschow D.J. (2018). ISG15 in Antiviral Immunity and Beyond. Nat. Rev. Genet..

[170] Freitas B.T., Scholte F.E., Bergeron É., Pegan S.D. (2020). How ISG15 Combats Viral Infection. Virus Res..

[171] Dzimianski J.V., Scholte F.E.M., Williams I.L., Langley C., Freitas B.T., Spengler J.R., Bergeron É., Pegan S.D. (2019). Determining the Molecular Drivers of Species-specific Interferon-stimulated Gene Product 15 Interactions with Nairovirus Ovarian Tumor Domain Proteases. PLoS ONE.

[172] Gori-Savellini G., Valentini M., Cusi M.G. (2013). Toscana Virus NSs Protein Inhibits the Induction of Type I Interferon by Interacting with RIG-I. J. Virol..

[173] Fan L., Briese T., Lipkin W.I. (2009). Z Proteins of New World Arenaviruses Bind RIG-I and Interfere with Type I Interferon Induction. J. Virol..

[174] Xing J., Ly H., Liang Y. (2015). The Z Proteins of Pathogenic but Not Nonpathogenic Arenaviruses Inhibit RIG-i-Like Receptor-Dependent Interferon Production. J. Virol..

[175] Martínez-Sobrido L., Zúñiga E.I., Rosario D., García-Sastre A., De La Torre J.C. (2006). Inhibition of the Type I Interferon Response by the Nucleoprotein of the Prototypic Arenavirus Lymphocytic Choriomeningitis Virus. J. Virol..

[176] Martínez-Sobrido L., Giannakas P., Cubitt B., García-Sastre A., De La Torre J.C. (2007). Differential Inhibition of Type I Interferon Induction by Arenavirus Nucleoproteins. J. Virol..

[177] Rodrigo W.W.S.I., Ortiz-Riaño E., Pythoud C., Kunz S., De La Torre J.C., Martínez-Sobrido L. (2012). Arenavirus Nucleoproteins Prevent Activation of Nuclear Factor Kappa B. J. Virol..

[178] Pythoud C., Rodrigo W.W.S.I., Pasqual G., Rothenberger S., Martinez-Sobrido L., De La Torre J.C., Kunz S. (2012). Arenavirus Nucleoprotein Targets Interferon Regulatory Factor-Activating Kinase IKK. J. Virol..

[179] Taylor S.L., Krempel R.L., Schmaljohn C.S. (2009). Inhibition of TNF-α-induced Activation of NF-κB by Hantavirus Nucleocapsid Proteins. Ann. N. Y. Acad. Sci..

[180] Ontiveros S.J., Li Q., Jonsson C.B. (2010). Modulation of Apoptosis and Immune Signaling Pathways by the Hantaan Virus Nucleocapsid Protein. Virology.

[181] Pan W., Bian G., Wang K., Feng T., Dai J. (2015). Effects of Different Doses of Nucleocapsid Protein from Hantaan Virus A9 Strain on Regulation of Interferon Signaling. Viral Immunol..

[182] Taylor S.L., Frias-Staheli N., García-Sastre A., Schmaljohn C.S. (2008). Hantaan Virus Nucleocapsid Protein Binds to Importin α Proteins and Inhibits Tumor Necrosis Factor Alpha-Induced Activation of Nuclear Factor Kappa B. J. Virol..

[183] Cimica V., Dalrymple N.A., Roth E., Nasonov A., Mackow E.R. (2014). An Innate Immunity-Regulating Virulence Determinant Is Uniquely Encoded within the Andes Virus Nucleocapsid Protein. MBio.

[184] Simons M.J., Gorbunova E.E., Mackow E.R. (2019). Unique Interferon Pathway Regulation by the Andes Virus Nucleocapsid Protein Is Conferred by Phosphorylation of Serine 386. J. Virol..

[185] Levine J.R., Prescott J., Brown K.S., Best S.M., Ebihara H., Feldmann H. (2010). Antagonism of Type I Interferon Responses by New World Hantaviruses. J. Virol..

[186] Alff P.J., Gavrilovskaya I.N., Gorbunova E., Endriss K., Chong Y., Geimonen E., Sen N., Reich N.C., Mackow E.R. (2006). The Pathogenic NY-1 Hantavirus G1 Cytoplasmic Tail Inhibits RIG-I- and TBK-1-Directed Interferon Responses. J. Virol..

[187] Alff P.J., Sen N., Gorbunova E., Gavrilovskaya I.N., Mackow E.R. (2008). The NY-1 Hantavirus Gn Cytoplasmic Tail Coprecipitates TRAF3 and Inhibits Cellular Interferon Responses by Disrupting TBK1-TRAF3 Complex Formation. J. Virol..

[188] Matthys V.S., Cimica V., Dalrymple N.A., Glennon N.B., Bianco C., Mackow E.R. (2014). Hantavirus GnT Elements Mediate TRAF3 Binding and Inhibit RIG-I/TBK1-Directed Beta Interferon Transcription by Blocking IRF3 Phosphorylation. J. Virol..

[189] Qu B., Qi X., Wu X., Liang M., Li C., Cardona C.J., Xu W., Tang F., Li Z., Wu B. (2012). Suppression of the Interferon and NF-κB Responses by Severe Fever with Thrombocytopenia Syndrome Virus. J. Virol..

[190] Santiago F.W., Covaleda L.M., Sanchez-Aparicio M.T., Silvas J.A., Diaz-Vizarreta A.C., Patel J.R., Popov V., Yu X.-J., García-Sastre A., Aguilar P.V. (2014). Hijacking of RIG-I Signaling Proteins into Virus-Induced Cytoplasmic Structures Correlates with the Inhibition of Type I Interferon Responses. J. Virol..

[191] Wu X., Qi X., Qu B., Zhang Z., Liang M., Li C., Cardona C.J., Li D., Xing Z. (2013). Evasion of Antiviral Immunity through Sequestering of TBK1/IKK /IRF3 into Viral Inclusion Bodies. J. Virol..

[192] Ning Y.-J., Wang M., Deng M., Shen S., Liu W., Cao W.-C., Deng F., Wang Y.-Y., Hu Z., Wang H. (2014). Viral Suppression of Innate Immunity via Spatial Isolation of TBK1/IKKε from Mitochondrial Antiviral Platform. J. Mol. Cell Biol..

[193] Hong Y., Bai M., Qi X., Li C., Liang M., Li D., Cardona C.J., Xing Z. (2019). Suppression of the IFN-α and -β Induction through Sequestering IRF7 into Viral Inclusion Bodies by Nonstructural Protein NSs in Severe Fever with Thrombocytopenia Syndrome Bunyavirus Infection. J. Immunol..

[194] Moriyama M., Igarashi M., Koshiba T., Irie T., Takada A., Ichinohe T. (2018). Two Conserved Amino Acids within the NSs of Severe Fever with Thrombocytopenia Syndrome Phlebovirus Are Essential for Anti-interferon Activity. J. Virol..

[195] Spiropoulou C.F., Albariño C.G., Ksiazek T.G., Rollin P.E. (2007). Andes and Prospect Hill Hantaviruses Differ in Early Induction of Interferon although Both Can Downregulate Interferon Signaling. J. Virol..

[196] Ning Y.-J., Feng K., Min Y.-Q., Cao W.-C., Wang M., Deng F., Hu Z., Wang H. (2015). Disruption of Type I Interferon Signaling by the Nonstructural Protein of Severe Fever with Thrombocytopenia Syndrome Virus via the Hijacking of STAT2 and STAT1 into Inclusion Bodies. J. Virol..

[197] Kainulainen M., Lau S., Samuel C.E., Hornung V., Weber F. (2016). NSs Virulence Factor of Rift Valley Fever Virus Engages the F-Box Proteins FBXW11 and β-TRCP1 To Degrade the Antiviral Protein Kinase PKR. J. Virol..

[198] Mudhasani R., Tran J.P., Retterer C., Kota K.P., Whitehouse C.A., Bavari S. (2016). Protein Kinase R Degradation Is Essential for Rift Valley Fever Virus Infection and Is Regulated by SKP1-CUL1-F-box (SCF)FBXW11-NSs E3 Ligase. PLoS Pathog..

[199] Kalveram B., Ikegami T. (2013). Toscana Virus NSs Protein Promotes Degradation of Double-Stranded RNA-Dependent Protein Kinase. J. Virol..

[200] Wang Z., Mir M.A. (2014). Andes Virus Nucleocapsid Protein Interrupts Protein Kinase R Dimerization to Counteract Host Interference in Viral Protein Synthesis. J. Virol..

[201] Gale M., Blakely C.M., Kwieciszewski B., Tan S.-L., Dossett M., Tang N.M., Korth M.J., Polyak S.J., Gretch D.R., Katze M.G. (1998). Control of PKR Protein Kinase by Hepatitis C Virus Nonstructural 5A Protein: Molecular Mechanisms of Kinase Regulation. Mol. Cell. Biol..

[202] Faburay B., LaBeaud A.D., McVey D.S., Wilson W.C., Richt J.A. (2017). Current Status of Rift Valley Fever Vaccine Development. Vaccines.

[203] Jégouic S., Joffret M.-L., Blanchard C., Riquet F.B., Perret C., Pelletier I., Colbère-Garapin F., Rakoto-Andrianarivelo M., Delpeyroux F. (2009). Recombination between Polioviruses and Co-Circulating Coxsackie A Viruses: Role in the Emergence of Pathogenic Vaccine-Derived Polioviruses. PLoS Pathog..

[204] Makoschey B., Van Kilsdonk E., Hubers W.R., Vrijenhoek M.P., Smit M., Schreur P.J.W., Kortekaas J., Moulin V. (2016). Rift Valley Fever Vaccine Virus Clone 13 Is Able to Cross the Ovine Placental Barrier Associated with Foetal Infections, Malformations, and Stillbirths. PLoS Negl. Trop. Dis..

[205] Scholte F.E.M., Hua B.L., Spengler J.R., Dzimianski J.V., Coleman-McCray J.D., Welch S.R., McMullan L.K., Nichol S.T., Pegan S.D., Spiropoulou C.F. (2019). Stable Occupancy of the Crimean-Congo Hemorrhagic Fever Virus-Encoded Deubiquitinase Blocks Viral Infection. MBio.

